# Polysaccharides utilization in human gut bacterium *Bacteroides thetaiotaomicron*: comparative genomics reconstruction of metabolic and regulatory networks

**DOI:** 10.1186/1471-2164-14-873

**Published:** 2013-12-12

**Authors:** Dmitry A Ravcheev, Adam Godzik, Andrei L Osterman, Dmitry A Rodionov

**Affiliations:** 1 Sanford-Burnham Medical Research Institute, La Jolla, California 92037, USA; 2 A.A. Kharkevich Institute for Information Transmission Problems, Russian Academy of Sciences, Moscow 127994, Russia

**Keywords:** Regulatory network, Regulon, Transcription factor, BACTEROIDES, Carbohydrate utilization

## Abstract

**Background:**

*Bacteroides thetaiotaomicron*, a predominant member of the human gut microbiota, is characterized by its ability to utilize a wide variety of polysaccharides using the extensive saccharolytic machinery that is controlled by an expanded repertoire of transcription factors (TFs). The availability of genomic sequences for multiple *Bacteroides* species opens an opportunity for their comparative analysis to enable characterization of their metabolic and regulatory networks.

**Results:**

A comparative genomics approach was applied for the reconstruction and functional annotation of the carbohydrate utilization regulatory networks in 11 *Bacteroides* genomes. Bioinformatics analysis of promoter regions revealed putative DNA-binding motifs and regulons for 31 orthologous TFs in the *Bacteroides*. Among the analyzed TFs there are 4 SusR-like regulators, 16 AraC-like hybrid two-component systems (HTCSs), and 11 regulators from other families. Novel DNA motifs of HTCSs and SusR-like regulators in the *Bacteroides* have the common structure of direct repeats with a long spacer between two conserved sites.

**Conclusions:**

The inferred regulatory network in *B. thetaiotaomicron* contains 308 genes encoding polysaccharide and sugar catabolic enzymes, carbohydrate-binding and transport systems, and TFs. The analyzed TFs control pathways for utilization of host and dietary glycans to monosaccharides and their further interconversions to intermediates of the central metabolism. The reconstructed regulatory network allowed us to suggest and refine specific functional assignments for sugar catabolic enzymes and transporters, providing a substantial improvement to the existing metabolic models for *B. thetaiotaomicron*. The obtained collection of reconstructed TF regulons is available in the RegPrecise database (http://regprecise.lbl.gov).

## Background

The microbial community of the human gut plays an essential role in human health and physiology, for instance, in the degradation of host-indigestible polysaccharides [1-3]. In spite of the amazing variability of the phenotypes, 90-99% of bacteria in the human gut microbiota belong to only two phyla, the *Firmicutes* and *Bacteroidetes*[4]. On the genus level, the *Bacteroides* spp. is the most abundant in the human bowel [5,6]. One of the most studied representatives of this genus is *Bacteroides thetaiotaomicron*, a Gram-negative obligate anaerobe that is notable for its ability to utilize a wide variety of polysaccharides [7,8]. In total, this microorganism can utilize dozens of dietary plant polysaccharides and host-derived mucosal glycans. Genes for polysaccharide utilization loci (PUL) are organized in over 80 PULs [9]. Existing genome-scale metabolic reconstructions of *B. thetaiotaomicron* include a comprehensive description of reactions constituting the central metabolism and genes involved in host interaction but do not cover peripheral sugar metabolism [10].

Polysaccharide utilization in *B. thetaiotaomicron* is characterized by specific features exhibited on three levels: enzymatic, transport, and regulatory. On the enzymatic level, the saccharolytic machinery in *B. thetaiotaomicron* includes a large number of extracellular and periplasmic proteins involved in polysaccharide binding, processing, and cleavage. According to the CAZy database (http://www.cazy.org) [11], *B. thetaiotaomicron* possesses 269 glycoside hydrolases, 87 glycosyl transferases, 15 polysaccharide lyases, and 19 carbohydrate esterases. After digestion by extracellular enzymes, oligosaccharides are imported into the periplasm by utilizing the sugar-specific outer-membrane systems of SusC/D. These systems were named after the first described representative, starch utilization system (SUS), which is responsible for the transport of products of starch digestion [12]. Homologs of two of the *sus* operon members, *susD* and *susC*, are present in every PUL. Proteins coded by these two genes, SusC-/SusD-like proteins, form a transport system consisting of an outer-membrane TonB-dependent porin (a SusC-like protein) and a glycan-binding SusD-like protein [13]. Overall, 101 individual pairs of genes coding for SusC-/D-like systems were detected in the *B. thetaiotaomicron* genome [14], forming 88 PUL systems (some PUL systems contain multiple SusC/D pairs).

Three types of *Bacteroidetes* specific regulatory systems were proposed to be involved in transcriptional regulation of genes for PUL in *B. thetaiotaomicron* and other *Bacteroides* spp.: (1) SusR-like regulators, (2) hybrid two-component systems (HTCSs), and (3) extracytoplasmic function (ECF) sigma/anti-sigma factors. The SusR protein was first described as a regulator of the starch utilization system in *B. thetaiotaomicron*. SusR is a membrane protein with a sensor domain exposed to the periplasm and a DNA binding domain located in the cytoplasm. In the presence of starch, the SusR protein binds to the promoters of the starch utilization genes organized in the operons *susA* and *susBCDEFG* and activates their expression [12]. HTCSs are chimeric proteins containing the cytoplasmic components of “classical” two-component systems: a transmembrane sensor histidine kinase and a DNA-binding response regulator, as a single polypeptide, which is fused to a large carbohydrate-sensing domain in the periplasm. Previously, 32 HTCSs were predicted in the *B. thetaiotaomicron* genome [9], and the carbohydrate-sensing domains of some of these proteins were experimentally analyzed [15]. The third type of *Bacteroides*-specific regulatory systems includes the ECF-family sigma factors and their cognate anti-sigma factors from the FrrF family possessing two domains connected by a transmembrane linker [16,17]. The periplasmic domains of anti-sigma factors interact with specific SusC-like carbohydrate porins, whereas the cytoplasmic domains interact with congruent ECF sigma factors. In the presence of corresponding glycans, sigma factors are released and activate the regulated operons. In total, 26 sigma/anti-sigma systems associated with the sugar utilization genes were identified in the *B. thetaiotaomicron* genome [18].

Glycans utilization in *B. thetaiotaomicron* has been intensively studied during the last 10 years [7,19]. However, the knowledge of sugar-specific metabolic and regulatory networks is still fragmentary and/or incomplete. Only a small subset of PUL transcriptional factors (TFs), including the SusR regulator and some HTCSs, has been characterized to date. Even for these well-studied regulators, while their polysaccharide specificities and sets of regulated genes have been identified, their DNA-binding sites remain unknown.

In this work, a comparative genomics approach combined with metabolic reconstruction was applied to infer regulatory networks for polysaccharide and sugar utilization genes in *B. thetaiotaomicron*. Previously, a similar combined approach was applied for regulatory network reconstruction in diverse lineages of both Gram-positive and Gram-negative bacteria [20-26]. In the absence of any experimental data about regulation, the subsystem-oriented strategy, which is based on the assumption that the genes from the same metabolic pathway may be regulated by one TF (as reviewed in [27]), could be used to identify novel TF regulons. The methods of *phylogenetic footprinting* and *consistency check* (described in Methods) are powerful approaches for discovery and characterization of microbial regulons. Here, we utilized these comparative genomic approaches to study the genomes of *B. thetaiotaomicron* and 10 other *Bacteroides* spp. As result, we report the identification of novel DNA binding motifs for numerous HTCS and SusR-like regulators, as well as for 11 other regulators from conventional TF families controlling the sugar metabolism. The inferred regulatory network in *B. thetaiotaomicron* allowed us to refine and improve the metabolic reconstruction of pathways for degradation of complex glycans to sugar monomers and the respective monosaccharide utilization pathways.

## Results and discussion

### Repertoire of the SusR-like and HTCS regulatory systems in *B. thetaiotaomicron*

To estimate the scale and diversity of the studied regulatory systems, we performed a genetic census of the putative HTCSs and SusR-like proteins in *B. thetaiotaomicron* and 10 related *Bacteroides* genomes (see “Methods”).

A search of paralogs for the *B. thetaiotaomicron* SusR (BT3705) protein revealed four additional SusR-like proteins (Table 1). All identified SusR paralogs in *B. thetaiotaomicron* have orthologs in other *Bacteroides* genomes. The BT3705 and BT3091 proteins are evolutionarily conserved in five and six genomes, respectively. Three other SusR paralogs (BT3309, BT2160, and BT4069) in *B. thetaiotaomicron* have orthologs only in the closely related genome of *B. ovatus*. The SusR family of regulatory proteins is a unique feature of the *Bacteroidetes* phylum, as other phyla of bacteria apparently lack SusR homologs.

**Table 1:** **SusR-like and HTCS regulators in *B. thetaiotaomicron***

**Locus tag**^**1**^	**Regulator name**^**2**^	**Orthologs**^**3**^	**Utilized polysaccharides**^**4**^
**HTCS regulators**			
BT0138		4	
BT0267	HTCS_Aga-1	1	Arabinogalactans^*b, c*^
BT0366	HTCS_Ara-1	6	Arabinans^*a, b*^
BT0958		2	
BT0981		6	Rhamnogalacturonans^*b, c*^
BT1635		2	N-Acetylglucosamine polymers^*c*^
BT1734		7	
BT1754	HTCS_Fru	10	Fructosides^*a, b*^
BT2391		1	
BT2628	HTCS_Man-1	3	Mannans^*b, c*^
BT2826	HTCS_Ogl-1	1	O-glycans^*b, c*^
BT2860		1	
BT2897		5	Arabinans^*c*^
BT2923		4	
BT2971		1	N-Acetylglucosamine polymers^*d*^
BT3049	HTCS_Ara-2	6	Arabinans^*b, c*^
BT3097		2	Arabinans^*c*^
BT3134		3	N-Acetylglucosamine polymers^*d*^
BT3172		2	Mannans^*a*^
BT3302	HTCS_Man-2	1	Mannans^*b, c*^
BT3334	HTCS_Hya	7	Chondroitin sulfate, Hyaluronan^*b, c*^
BT3465		3	
BT3678		1	Arabinans^*c*^
BT3738		3	
BT3786	HTCS_Man-3	4	Mannans^*b, c*^
BT3800		3	N-Acetylglucosamine polymers^*c*^
BT3951		3	
BT3957		3	Mannans^*c*^
BT4111	HTCS_Rgu-1	8	Rhamnogalacturonans^*b, c*^
BT4124	HTCS_Rgu-1	2	Rhamnogalacturonans^*b, c*^
BT4137	HTCS_Ogl-2	1	O-glycans^*b, c*^
BT4178	HTCS_Rgu-2	3	Rhamnogalacturonans ^*b, c*^
BT4182	HTCS_Rgu-2	2	Rhamnogalacturonans ^*b, c*^
BT4236		2	
BT4663	HTCS_Hep	4	Heparin^*a, b*^
BT4673	HTCS_Pga	3	Pectic galactan^*b*^
**SusR-like regulators**			
BT2160	SusR4	2	
BT3091	SusR2	5	Dextran ^*b, c*^
BT3309	SusR3	2	
BT3705	SusR	6	Starch^*a*^
BT4069		2	

For details, see Additional file 1. ^1^Regulator locus tags are shown for B. *thetaiotaomicron* VPI-5482. ^2^Regulator names are shown for only the regulons reconstructed in this study. ^3^Number of regulator’s orthologs found in 11 analyzed genomes of *Bacteroides* spp.^4^Superscript letters show different types of evidence for the predicted polysaccharide utilization pathway that is controlled by studied regulators: (a) *in vitro* glycan binding to the sensor domain of a regulator; (b) differential expression of sugar utilization genes that are located in the same chromosomal cluster with a regulator gene [28]; (c) bioinformatics analysis of co-localization of a regulator gene with sugar utilization genes.

Protein similarity searches revealed 36 genes encoding HTCSs in the *B. thetaiotaomicron* genome (Table 1). Eight of these HTCS paralogs are species-specific among the strains analyzed and were found only in *B. thetaiotaomicron*, whereas the remaining HTCS proteins have from one to nine orthologs in other analyzed *Bacteroides*. A smaller number of HTCS homologs were identified in several other lineages from the *Bacteroidetes* phylum including *Flavobacteriales*, *Cytophagales*, and *Sphingobacteriales*. Similarly to the SusR family, the HTCS family is a unique feature of the *Bacteroidetes* phylum.

All identified HTCS proteins have a characteristic domain structure containing an N-terminal periplasmic ligand-binding sensor domain, a cytoplasmic signal transduction histidine kinase domain, and a C-terminal response regulator containing a CheY-like receiver domain and an AraC-type DNA-binding domain. Most HTCSs in *B. thetaiotaomicron* have the periplasmic sensor domain belonging to the COG3292. The only exception is the fructose-sensing HTCS encoded by the *BT1754* gene, which has a different sensor domain from the COG1879 family shared with periplasmic components of ABC-type sugar transporters. Also, BT1754 is the only known HTCS sensing sugar monomers [9], whereas all studied HTCSs with the COG3292 sensor domain are known to bind oligosaccharides [15,28]. Thus, we can propose that the sensor domain family determines the type of sugars (mono-or oligosaccharides) recognized by the HTCS regulators.

### Functional context analysis for the SusR-like and HTCS regulatory systems in *B. thetaiotaomicron*

To predict biological roles of SusR-like and HTCS regulators, we analyzed functions of genes that are co-localized on the chromosome with a regulatory gene and their orthologs across all *Bacteroides* genomes. In addition to the analysis of conserved chromosomal gene clusters, we used differential gene expression data available for *B. thetaiotaomicron* for functional annotation of genes upregulated on specific polysaccharides [28].

Genes encoding 25 HTCSs and 3 SusR-like regulators are co-localized on the chromosome with the *susC-/susD*-like genes in *B. thetaiotaomicron* (Additional file 1). The SusC-/SusD-like systems are involved in the binding and uptake of oligosaccharides into the periplasmic space [16]. Thus, the identified regulators genomically associated with these systems are most likely involved in the transcriptional control in response to their respective oligosaccharides. The remaining 11 HTCSs and 2 SusR-like regulators are not linked to the *susC-/susD*-like genes in the genome, raising the question of their involvement in the control of polysaccharide and sugar utilization (PSU) metabolism. Among the latter group of regulators, four HTCS genes and one *susR*-like gene are co-localized with one or multiple genes encoding saccharolytic enzymes, suggesting their involvement in a PSU pathway regulation in the absence of a cognate SusC/SusD system. The remaining regulators that lack both *susC/susD*-like and PSU genes in their genomic neighborhoods may potentially regulate distant genomic loci; however, identification of their target genes and effectors will require future experiments.

Among the five SusR-like regulators in *B. thetaiotaomicron*, only the starch utilization regulator SusR (BT3705) was previously functionally characterized [12]. Based on the conserved co-localization with genes encoding dextranase and glucan 1,3-α-glucosidase, we predicted that SusR2 (BT3091) likely functions as a regulator for the dextran utilization pathway. During the preparation of this article for publication, the prediction of BT3091 function as a regulator of dextran utilization was confirmed by an independent research group. Thus, expression of the SusC homolog BT3090 was shown to be dextran induced [29]. Genes encoding two other SusR-like proteins, *BT3309* and *BT2160*, are co-localized with predicted glycosyl hydrolases of yet-unknown specificities, whereas the *BT4069* gene has no conserved chromosomal neighbors. Thus, functions of the latter three SusR-like regulators could not be predicted via the chromosomal context analysis.

Among 36 HTCSs, ligand specificity of the sensor domains has been experimentally determined for only four regulatory systems: BT0366 (oligoarabinan), BT1754 (fructose), BT3172 (mannosides), and BT4663 (heparin) [9,15,30]. In agreement with these data, genes encoding three of these HTCSs are located within the chromosomal clusters involved in the utilization of arabinan, fructosides, and heparin. Using available data on the differential gene expression in *B. thetaiotaomicron* grown on different polysaccharides [28] and/or the genomic context analysis, we predicted candidate functional roles for the other 22 HTCS regulators (Table 1). Functions of eight of these regulators were predicted by using only the genomic context analysis. Finally, functional roles of 10 HTCSs in *B. thetaiotaomicron* remain unknown.

### Analysis of SusR-like regulons

SusR-like proteins are unique and poorly studied regulators. The SusR protein from *B. thetaiotaomicron* was shown to bind maltose in the periplasm and activate expression of two operons, *susA* and *susBCDEFG*[12]. The C-terminal DNA binding domain in SusR has no significant similarity to DNA binding domains of other known TFs; however, a putative helix-turn-helix motif presumably involved in DNA binding has been identified near its carboxy terminus [12]. Nonetheless, DNA binding sites for SusR remained uncharacterized. Based on our previous analyses of DNA binding motifs of regulators from several TF families [21,31-33], we proposed that binding motifs for homologous SusR-like regulators in *Bacteroides* may appear similar to each other. Thus, for prediction of SusR regulatory motifs we used the following pipeline: (1) genes known or predicted to be co-regulated by SusR-like proteins were selected by genome context analysis in all *Bacteroides* genomes, (2) conserved DNA motifs were predicted in upstream regions of candidate co-regulated operons using a phylogenetic footprinting approach, and (3) the identified motifs were compared to each other and assigned to SusR-like regulators.

**Figure 1:**
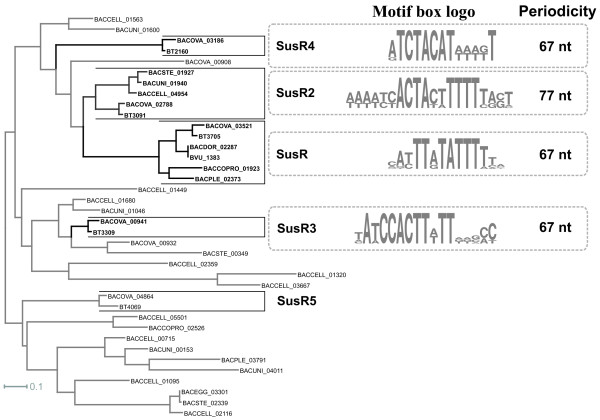
**Comparison of the motifs for the reconstructed regulons for SusR-like proteins.** Maximum-likelihood phylogenetic tree for the predicted HTH domains of the SusR-like proteins in studied genomes and sequence logos for the predicted binding DNA-motifs. SusR-like proteins with reconstructed regulons are shown in bold.

A search for SusR homologs in 11 studied *Bacteroides* genomes revealed, in total, 38 SusR-like proteins (Figure 1). Phylogenetic analysis of their C-terminal DNA binding domains allowed us to refine the predicted orthologous groups for five SusR-like proteins from *B. thetaiotaomicron* (Figure 1). Analysis of the regulatory regions of genes regulated by SusR-like proteins allowed us to identify putative regulatory motifs for the “canonical” regulator SusR (BT3705) and three other regulators: SusR2 (BT3091), SusR3 (BT3309), and SusR4 (BT2160). Each predicted DNA motif has a direct repeat structure with two conserved sites separated by a long non-conserved spacer (Additional file 2). The predicted SusR motifs are characterized by an unusually long distance between the centers of tandem sites (67 or 77 bp). The structure of the predicted binding motifs suggests that SusR-like proteins bind DNA as dimers, where the monomers sit on the same side of DNA and are separated by six or seven helix turns. Such a long distance between two boxes can be explained by the large size of the SusR-like proteins. The similarity of the predicted binding motifs correlates with the similarity of the regulators themselves (Figure 1). Thus, the highest similarity was observed between binding motifs for SusR and SusR2 regulators that form proximal branches on the phylogenetic tree.

Functions of the regulated genes were previously characterized for the starch utilization regulon SusR [13]. The SusR2 regulon is involved in utilization of the glucose polymer dextran, as it contains genes encoding the SusC-/SusD-like proteins, a SusE homolog, and glucan 1,3-alpha-glucosidase and dextranase (Additional file 3). The SusR3 and SusR4 regulons are involved in the utilization of a yet-unknown polysaccharide, as both regulons contain genes for hypothetical glycosyl hydrolases of unknown specificity.

### Analysis of HTCS regulons

**Figure 2:**
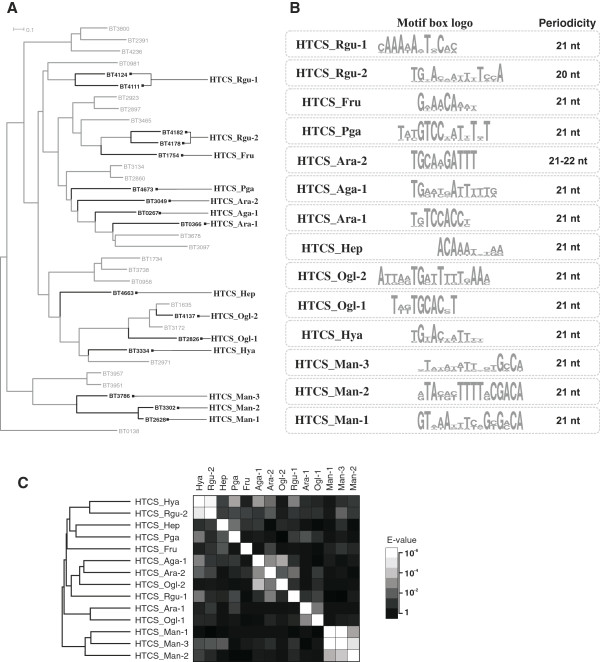
**Comparison of the motifs for the reconstructed regulons for HTCS regulatory systems. (A)** Maximum-likelihood phylogenetic tree for the HTH domains of B. *thetaiotaomicron* HTCSs; HTCSs with reconstructed regulons are shown in bold (the tree for all HTCSs in the studied genomes is shown in Additional file 4: Figure S1). **(B)** Logos for the predicted DNA binding motifs. **(C)** Heat map of e-values for the predicted HTCS binding motifs.

A total of 36 HTCS paralogs were detected in the *B. thetaiotaomicron* genome. All HTCS regulators have a C-terminal DNA binding domain containing a helix-turn-helix motif of the AraC type. Although carbohydrate-binding specificities were previously characterized for four HTCS systems, the DNA binding motifs recognized by HTCSs were not known before this work. To identify candidate DNA motifs and reconstruct regulons for HTCS systems, we used the comparative genomics pipeline similar to that used for the SusR regulons. To enable this approach, only those *B. thetaiotaomicron* HTCSs that satisfied the following criteria were analyzed: (1) orthologs of an HTCS are present in related genomes, according to the phylogenetic tree of all HTCS homologs found in *Bacteroides* spp. (Figure 2 and Additional file 4: Figure S1); (2) an HTCS gene is co-localized on the chromosome with *susC-/susD*-like genes (Additional file 3); and (3) genes from the PSU locus presumably regulated by HTCS are differentially expressed on a specific polysaccharide (Additional file 1).

As a result, we predicted DNA binding motifs and reconstructed regulons for 16 out of 36 HTCSs in *B. thetaiotaomicron* (Figure 2A). All predicted DNA motifs have the similar structure of a direct repeat with two conserved sites separated by a non-conserved spacer (Figure 2B). The average distance between tandem sites in the predicted HTCS binding motifs was 21 bp. The structure of the predicted binding motifs suggests that HTCS proteins bind DNA as dimers, where the monomers sit on the same side of DNA and are separated by two helix turns. Pairwise comparison and clustering of the identified HTCS binding motifs were performed to assess correlations between DNA binding domains in HTCSs and their cognate DNA motifs (Figure 2C). This analysis revealed several groups of HTCS paralogs with similar DNA motifs that generally correspond to the clusters of respective HTCS proteins on the phylogenetic tree (Figure 2A). For instance, three HTCS_Man regulators (BT3786, BT3302, and BT2628) form a monophyletic branch on the tree, and their corresponding DNA motifs are also clustered with each other, demonstrating a high level of pairwise similarity (Figure 2C).

**Figure 3:**
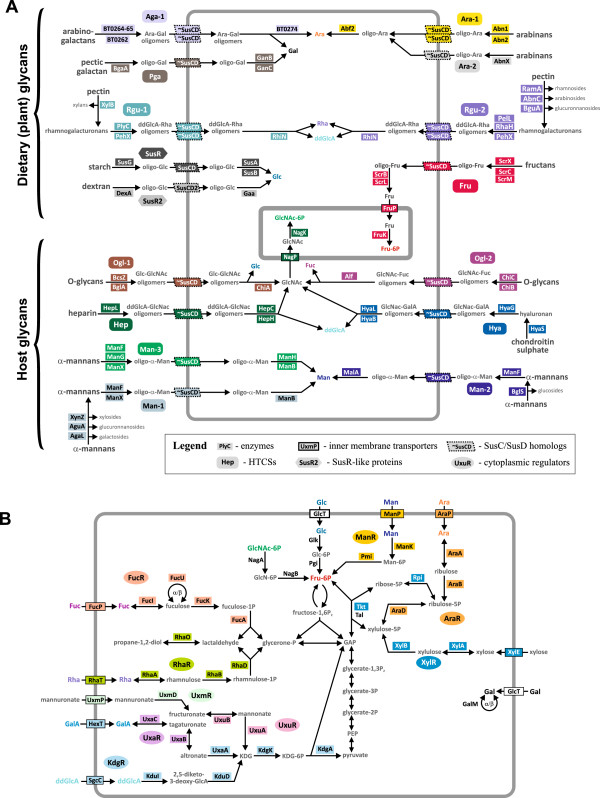
**Reconstructed metabolic and regulatory pathways in *B. thetaiotaomicron*. (A)** pathways for utilization of dietary and host glycans; **(B)** pathways for utilization of monosaccharides in the cytoplasm. Regulators and proteins from the corresponding regulons are shown by matching background colors. Abbreviations for monosaccharides: Ara, L-arabinose; ddGlcA, 5-dehydro-4-deoxy-D-glucuronate; Fuc, L-fucose; Fru, D-fructose; Gal, D-galactose; GalA, galacturonate; Glc, D-glucose; GlcA, glucuronate; GlcNAc, N-acetyl-D-glucosamine; KDG, 2-keto-3-deoxy-D-gluconate; Man, D-mannose; Rha, L-rhamnose.

Functional and metabolic content of the reconstructed HTCS regulons in *B. thetaiotaomicron* are provided in Additional file 3 and are also visualized on the integrative *B. thetaiotaomicron* PSU network (Figure 3 and Additional file 5). Regulon content for orthologous HTCSs in other *Bacteroides* genomes is available in the RegPrecise database [34]. Below, we describe the reconstructed regulatory network for three polysaccharide utilization pathways in more detail.

Heparin and heparan sulfate are host-derived glycans containing N-acetylglucosamine monosaccharides that can be utilized by *B. thetaiotaomicron*[35]. The heparin-induced gene locus *BT4652-62*[28] encodes the experimentally studied HTCS system, BT4663, whose sensor domain is able to bind heparin and heparan sulfate [15]. Since BT4663 is associated with the heparin and heparan sulfate utilization genes, we named this regulator HTCS_Hep. We analyzed the promoter regions for the respective heparin-induced genes and identified a common direct repeat motif upstream of the *BT4662* and *BT4675* genes conserved in four *Bacteroides* genomes (Additional file 2). A similar DNA motif was also identified upstream of the *BT4658-52* operon, and it was conserved in the *B. ovatum* genome. As a result, the reconstructed HTCS_Hep regulon includes all genes from the heparin-induced gene locus organized into two operons (*BT4658-52* and *BT4662-59*), as well as the distantly localized gene *BT4675*. The inferred HTCS_Hep regulon includes genes for enzymes and transporters that together constitute the pathway for utilization of heparin or heparan sulfate to N-acetylglucosamine (Figure 3), whereas *BT4675* encodes the periplasmic heparin lyase I/heparinase I (HepC).

Alpha-mannans are common components of human-derived N-glycans, where mannose monomers form a mannose-rich core [36]. Thus, alpha-mannans can be used for the identification of genes induced by host N-glycans and enabled for utilization of these polysaccharides. Two PSU gene loci in *B. thetaiotaomicron* activated by alpha-mannans [28] encode two HTCSs, BT2628 and BT3786 (named HTCS_Man-1 and HTCS_Man-3). These two mannan-induced HTCSs are clustered on the phylogenetic tree (Figure 2A), and the respective cluster contains yet another HTCS paralog, BT3302 (named HTCS_Man-2). A search of DNA motifs revealed direct repeats of two 16-bp sites separated by a 5-bp spacer. The predicted motifs for three alpha-mannan-specific HTCSs demonstrate strong similarity to each other (Figure 2B and C). We propose that each HTCS_Man paralog regulates adjacent genes preceded by a candidate DNA motif. Some components of the HTCS_Man regulons are common, such as periplasmic and extracellular mannosidases and SusC/SusD transport systems, whereas the others are exclusive for individual regulons, such as xylanase, glucuronidase, and galactosidase in the HTCS_Man-1 regulon and beta-glucohydrolase in the HTCS_Man-2 regulon. Thus, three similar HTCS_Man systems control pathways for the utilization of different types of alpha-mannans.

Rhamnogalacturonans are polysaccharides forming main chains of pectin, the major component of plant cell walls [37,38]. Previously, expression of four PSU gene loci in *B. thetaiotaomicron* was demonstrated to be activated by rhamnogalacturonans [28]. We found that three of these loci enclose one or two HTCS paralogs. The *BT4108-23* locus encodes two HTCSs, BT4111 and BT4124, whereas two other HTCSs, BT4178 and BT4182, are associated with the *BT4145-83* locus. Both these loci also encode SusC/SusD homologs. The third rhamnogalacturonan-induced locus, *BT0977-1030*, contains a single HTCS (BT0981) and homologs of SusC/SusD transporters. Unfortunately, we were unable to predict a binding motif for this HTCS because this locus is not conserved between the analyzed genomes. Thus, we analyzed candidate DNA motifs for two pairs of HTCSs from the first two rhamnogalacturonan-induced loci.

BT4111 and BT4124 are closely related paralogs that form a single branch on the phylogenetic tree (Figure 2), suggesting that the *BT4108-23* gene locus is regulated by both HTCSs that can potentially bind to the same DNA sites. Thus, we named the common regulon controlled by BT4111 and BT4124 as HTCS_Rgu-1. By analyzing the promoter regions of the predicted operons in this locus, we identified conserved direct repeats of two 12-bp sites separated by a 9-bp spacer (Additional file 2). The HTCS_Rgu-1 regulon includes genes required for pectin utilization (Figure 3A). Sidechains of pectin are detached, and central chains are split to oligomeric fragments by a set of extracellular hydrolases and pectin lyases, encoded by genes of this regulon. Also, this regulon includes genes for the esterase that cuts acetyl and methyl groups from the carbohydrates of the main chain. Two SusC-/SusD-like systems import sugar oligomers into the periplasm, where the oligosaccharides are finally hydrolyzed to monosaccharides (Figure 3A). In addition, the HTCS_Rgu-1 regulon contains also one distant operon, *BT4187-85*, encoding enzymes for the extracellular detachment of xylose sidechains and cleavage of the main chain.

A similar situation was observed for two other rhamnogalacturonan-specific HTCSs, BT4178 and BT4182, which are encoded within the same PSU gene locus and are also clustered with each other on the phylogenetic tree of HTCS paralogs (Figure 2). Thus, for these two HTCS paralogs, we also proposed the existence of one DNA binding motif and a joint regulon, named HTCS_Rgu-2. The predicted motif for these regulators is a direct repeat of two 14-bp sites separated by a 6-bp spacer (Additional file 2). The reconstructed HTCS_Rgu-2 regulon includes large extracellular machinery for detaching pectin side chains and deacetylation and splitting its main chain; a SusC/SusD-like transport system; and periplasmic rhamnogalacturonyl hydrolases (Figure 3A). This regulon also contains the *BT2680* gene for possible beta-galactosidase, which is localized distantly from the other regulated genes.

### Common and specific features of metabolic pathways regulated by HTCSs and SusR-like proteins

Comparison of the reconstructed pathways regulated by 2 SusR-like proteins and 16 HTCSs revealed several characteristic features of these pathways: (i) all reconstructed pathways start from the extracellular polysaccharides that are degraded to oligomers by exported enzymes, (ii) the obtained oligosaccharides are imported into periplasm by SusC-/SusD-like systems, and (iii) the imported oligosaccharides are cleaved to monosaccharides in the periplasm. The only exception from this pattern is the HTCS_Ara-2 regulon that lacks periplasmic sugar-splitting enzymes.

Some components of the reconstructed regulons were found in a subset of regulons, whereas other components appeared to be unique for the certain regulons (Additional file 3). The presence of glycosyl hydrolases for removal of sidechains in glycans is a feature of the rhamnogalacturonan- and mannan-specific regulons (Figure 3). Esterases for deacetylation and/or demethylation of glycans were found only in the rhamnogalacturonan regulons. Sulfatases were observed in the hyaluronan-and heparin-specific regulons. In the hyaluronan utilization pathway, sulfatase is extracellular and ought to desulfate polymeric glycans, whereas in the heparin utilization pathway, sulfatase is a periplasmic protein hydrolyzing N-acetylglucosamine sulphate. Lyases, either periplasmic or extracellular, were observed in the rhamnogalacturonan-, heparin-, and hyaluronan-specific regulons. All these glycans contain either D-glucuronate or D-galacturonate residues, and the presence of lyases in these regulons suggests formation of a 5-dehydro-4-deoxy-D-glucuronate intermediate as one of the final products of these metabolic pathways.

Only two reconstructed regulons, HTCS_Hep and HTCS_Fru, include inner-membrane transporters and cytoplasmic kinases. Thus, pathways under the control of these HTCSs produce cytoplasmic sugar phosphates: fructose 6-phosphate and N-acetylglucosamine 6-phosphate, respectively. The other reconstructed pathways for HTCS regulons finally produce various monosaccharides in the periplasm (Figure 3A). To be exploited, these monosaccharides should be transported into the cytoplasm and further catabolized via committed metabolic pathways. Thus, we further analyzed the cytoplasmic sugar utilization pathways and reconstructed 11 TF regulons controlling these pathways (Table 2).

**Table 2:** **Reconstructed regulons for utilization of monosaccharides in *B. thetaiotaomicron***

**Regulator name**^**1**^	**Locus tag**^**2**^	**Regulator orthologs**^**3**^	**Sugar utilization pathways controlled by regulon**	**Regulator family**	**References**
AraR^*^	BT0354	8	Arabinose, arabinan	NrtR	
Crp	BT4338	11	Multiple sugars	Crp	
FucR	BT1272	7	Fucose	GntR	[41]
KdgR^*^	BT0487	10	Glucuronate, galacturonate	LacI	
ManR^*^	BT2103	3	Mannose, mannosides	GntR	
NanR^*^	BT0433	11	Sialic acid; N-acetylglucosamine	ROK	
RhaR	BT3768	8	Rhamnose	AraC	[39,40]
UxaR^*^	BT0824	6	Glucuronate, galacturonate	LacI	
UxmR^*^	BT3613	6	Mannuronate	LacI	
UxuR^*^	BT1434	3	Glucuronate	LacI	
XylR^*^	BT0791	10	Xylose	NrtR	

^1^Novel regulators identified in this research are marked by asterisks. ^2^Regulator locus tags are shown for *B. thetaiotaomicron* VPI-5482. ^3^Number of regulator’s orthologs found in 11 analyzed genomes of *Bacteroides* spp.

### Reconstruction of metabolic and regulatory pathways for monosaccharide utilization

The metabolic pathways for utilization of rhamnose [39,40] and fucose [41], as well as their respective transcriptional regulators RhaR and FucR, have been previously described; however, a DNA binding motif was determined for FucR [41] but not for RhaR. In the present work, we predict the FucR and RhaR binding motifs and describe the corresponding regulons (Additional file 3). Each reconstructed regulon includes a single target operon encoding a sugar transporter and a complete set of cytoplasmic enzymes for the respective monosaccharide catabolic pathway. Both fucose and rhamnose catabolic pathways are characterized by the same two metabolic products, lactaldehyde and glycerone phosphate, that are further utilized via the central glycolytic pathways (Figure 3B).

To reconstruct the pathways and regulons involved in utilization of other monosaccharides produced by the SusR- and HTCS-regulated pathways, we used the following strategy. For every monosaccharide, we searched for homologs of known sugar catabolic enzymes in the *B. thetaiotaomicron* genome using SEED [42,43] and KEGG [44,45] and for the existing metabolic reconstruction of *B. thetaiotaomicron*[10]. The pathway was classified to be completely reconstructed if all enzymes required for conversion of a sugar to an intermediate of the central metabolism were identified. For completely reconstructed pathways, we attempted to predict cognate TFs that were physically adjacent on the genome, and describe the corresponding TF regulons (see Methods). For some pathways, the missing genes were predicted via genomic analysis of conserved gene neighborhoods and reconstructed regulons.

**Figure 4:**
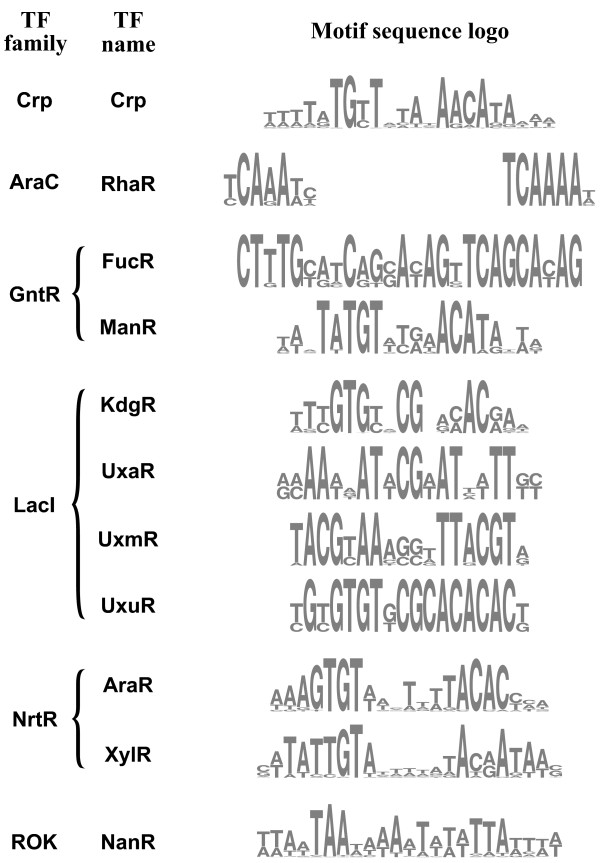
Sequence logos for the TFs regulating cytoplasmic monosaccharide utilization and for the global Crp-like regulator.

In addition to FucR and RhaR regulons, a total of eight novel TF regulons for the control of specific monosaccharide utilization pathways were predicted (Table 2, Figure 4). Most of the predicted regulators belonged to the protein families whose members were known to regulate pathways for carbohydrate metabolism, such as the AraC, GntR, LacI, and ROK families [46-49]. Two novel regulators for the arabinose and xylose utilization pathways, AraR (BT0354) and XylR (BT0791), belonged to the NrtR family of regulators that were demonstrated to control NAD biosynthesis in other bacterial phyla [50,51]. The experimental characterization of the NrtR-like sugar-responsive regulators in *B. thetaiotaomicron* is currently underway and will be published elsewhere (D.A. Rodionov, unpublished).

All reconstructed pathways for monosaccharide utilization include cytoplasmic enzymes and sugar transporters located in the inner membrane (Figure 3B). However, a limited number of genes encoding for periplasmic or extracellular proteins were identified in the AraR, KdgR, ManR, NanR, and UxmR regulons. The ManR regulon includes genes required for mannose uptake into cytoplasm and its further transformation to fructose 6-phosphate. In addition, the mannose utilization regulon contains the *BT2107-13* operon encoding extracellular oligo-α-mannosidases and a SusC-/SusD-like transport system. Thus, ManR regulates the complete pathway for degradation of extracellular α-mannosides to the central metabolism (Additional file 5). In addition to this ManR-controlled pathway, three other pathways and HTCS-type regulons were described for the mannan and α-mannoside utilization (see above). Such redundancy of the α-mannoside utilization metabolic and regulatory pathways can be explained by the variability of the α-mannosides produced by a host organism. The arabinose regulator AraR also co-regulates cytoplasmic and extracellular/periplasmic components of the PSU pathway. In addition to arabinose catabolic enzymes and arabinose transporters, the AraR regulon in *B. thetaiotaomicron* includes the *BT0365-60* operon encoding the endo-arabinosidase Abn1 and two pairs of SusC-/SusD-like proteins. Interestingly, the latter operon is under the dual control of AraR and HTCS_Ara-1 regulators.

The reconstructed metabolic and regulatory pathways for monosaccharide utilization build bridges between the extracellular/periplasmic pathways of degradation of host-and diet-derived complex glycans and the cytoplasmic pathways that feed digested monosaccharides into the central metabolism. However, the obtained metabolic and regulatory network is still incomplete as it reflects only some aspects of the *B. thetaiotaomicron* metabolism and transcriptional regulation and needs to be expanded and refined in the future.

### A putative global regulon for sugar utilization genes

The phylogenetic footprinting analysis of upstream regions of the *araMPRDAB*, *xylRBAE*, and *fucRIAKUP* operons regulated by AraR, XylR, and FucR, respectively, has revealed an additional conserved DNA motif (Additional file 2). The detected novel motif is a palindrome with consensus wwwTATGTTnTAnAACATAwww (where “w” stands for A or T) that is similar to the binding motif of the cAMP-responsive catabolic repressor protein Crp from *Escherichia coli*[52]. A genomic search for additional putative sites using this DNA motif and their comparative genomics analysis in 11 *Bacteroides* genomes revealed a putative global regulon containing up to 30 carbohydrate utilization genes per genome. In addition to the three above-mentioned sugar utilization operons, the novel regulon includes genes for utilization of galacturonate (*uxuRBA*), nucleosides (*nupG*), arabinosides (*BF0317*), β-hexosamines (*hex*), and pectin (*BT4112*), as well as pyruvate formate lyase (*pflBA*) and some other genes of unknown function (Additional file 3). Interestingly, a similar putative binding site was identified upstream of the *BT4338* gene encoding a hypothetical Crp-family transcription factor and its orthologs in ten other *Bacteroides* spp, suggesting possible autoregulation. Based on these genomic observations, we tentatively assigned BT4338 (termed Crp) as a putative transcription factor for the identified sugar catabolic regulon in *Bacteroides* spp. that binds to the identified 22-bp palindromic motif and activates gene expression in response to a yet-unknown cellular metabolite.

### Updated metabolic network for HTCSs and SusR-like regulons

Among 308 genes from the reconstructed PSU metabolic/regulatory network in *B. thetaiotaomicron*, 174 genes encode sugar catabolic enzymes, 65 genes encode proteins involved in sugar uptake (including 21 SusC/D-like systems), and 32 genes encode transcriptional regulators. For the remaining 37 genes we can predict only their general involvement in the particular PSU pathway; however, their precise functions are still undefined (Additional file 3). For example, the hypothetical gene *BT3047* belongs to the HTCS_Ara-2 regulon, and, thus, it is likely involved in the arabinan utilization pathway.

**Figure 5:**
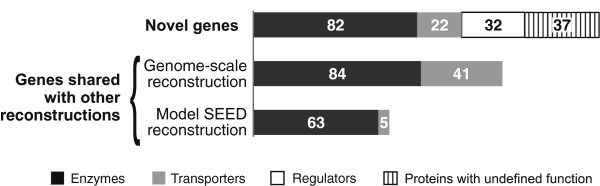
**Comparison of the reconstructed metabolic network with other metabolic models of *B. thetaiotaomicron.*** Top bar shows the number of novel genes in the reconstructed metabolic network in comparison with the previous genome-scale reconstruction in *B. thetaiotaomicron*[10] and with automatically generated metabolic reconstruction using the Model SEED tool [53], whereas the other two bars show numbers of genes that are shared between these reconstructions.

To evaluate the degree of novelty of our metabolic reconstruction, we compared our metabolic network with other metabolic models for the *B. thetaiotaomicron*. First, we used the Model SEED tool [53] for automated generation of a genome-based model for *B. thetaiotaomicron*. Second, we analyzed the manually curated genome-scale model for the *B. thetaiotaomicron* metabolism [10]. As a result, only 68 genes (encoding 63 enzymes and 5 transporters) were found to be common between our PSU network reconstruction and the metabolic model generated by Model SEED. Comparison of our reconstruction with the manually curated metabolic model demonstrated a larger overlap in 125 genes. Thus, the 84 enzyme genes and 41 transporter genes are shared between these two reconstructions (Figure 5). Overall, 55 enzyme genes and 3 transporter genes were shared between all three reconstructions.

The updated metabolic network in *B. thetaiotaomicron* contains 173 novel genes that were not included in any previous metabolic reconstruction. This expanded set of PSU genes encodes 82 enzymes, 22 transporters, 32 transcription factors, and 37 proteins of undefined functions. Thus, the usage of regulatory network reconstruction allowed us to expand the existing metabolic models.

Besides the addition of novel genes to the existing metabolic models, our metabolic reconstruction also includes the correction and improvement of the existing functional annotations for the genes. For example, in the Model SEED based metabolic model, the *BT3299* gene product was annotated as alpha-glucosidase. Our analysis of the pathways and regulons revealed that this gene encodes a periplasmic protein that belongs to the HTCS_Man-2 regulon. In agreement with the function of this regulon and predicted subcellular localization of the protein, the *BT3299* gene most probably encodes alpha-mannosidase. On the other hand, we performed numerous adjustments of functional annotations for genes from the previously published metabolic model [10]. Thus, in the previous model multiple genes from the *BT4108-23* gene locus were attributed to the rhamnogalacturonan degradation. In the current research, we assigned precise functional annotation for all enzymatic and transport genes from this locus and reconstructed the corresponding metabolic pathway, including the subcellular localization of the pathway steps.

The comparison of our metabolic network with the other metabolic models demonstrates that the usage of reconstruction of regulation for functional annotation and reconstruction of metabolism is a beneficial strategy. Owing to this approach we could adjust the existing functional annotations of genes that are involved in existing metabolic models and also expand the existing reconstruction by the large number of novel genes.

## Conclusions and future perspectives

The human gut bacterium *B. thetaiotaomicron* uses an arsenal of enzymes and transporters to degrade and utilize a variety of complex glycans including both host-synthesized glycans and diet polysaccharides resistant to host-mediated degradation [16]. In this work, we analyzed the repertoire of *B. thetaiotaomicron* transcription factors that are potentially involved in the control of genes involved in utilization of host and dietary complex polysaccharides systems, inferred the respective regulatory and metabolic networks using the comparative genomics approaches. The reconstructed regulatory network is operated by two types of membrane-anchored regulators that are unique to *Bacteroides*, namely the SusR-like proteins and HTCSs, as well as by a set of cytoplasmic regulators that belong to several other TF families. Overall, the obtained regulatory network in *B. thetaiotaomicron* contains ~290 genes, 30 local carbohydrate-responsive TFs, and a global Crp-like regulator. These numbers constitute ~6% of all genes and ~12.5% of all putative TFs encoded in the *B. thetaiotaomicron* genome. For the first time, we reported identification of putative DNA motifs for SusR-like regulators and HTCSs. The inferred metabolic network for genes from the analyzed TF regulons includes pathways for utilization of at least 12 major types of polysaccharides and 10 monosaccharides. The obtained metabolic network supplements and expands the previously published metabolic model of *B. thetaiotaomicron*[10] by providing novel functional annotations for ~100 genes from 12 peripheral sugar utilization pathways. Among these genes with newly predicted functions there are 30 SusC-/SusD-like protein pairs, a unique feature of the *Bacteroidetes*.

The resulting reconstruction of metabolism and regulatory interactions provides multiple opportunities for further research of PSU pathways and regulons in *B. thetaiotaomicron*. The repertoire of *B. thetaiotaomicron* TFs contains one SusR-like protein and 20 HTCSs for which we were unable to predict their cognate DNA motifs. Also, the *Bacteroidetes* genomes encode numerous ECF-family sigma-factor/anti-sigma systems that may potentially control PSU pathways. The prediction of ECF-controlled promoters is a challenging task that requires experimental data about the transcriptional start sites positions. Finally, the predicted DNA motifs and carbohydrate specificities for SusR-like proteins and HTCSs should be tested experimentally. Another prospective use of current reconstruction is to guide the field of structural bioinformatics. At this point, experimental protein structures are available for sensor domains of four HTCSs and for four SusD-like proteins. Sugar specificities were known for some of these proteins. In this research, we predicted sugar-binding specificities for all mentioned proteins and for a large numbers of their homologs. Thus, the results of our reconstruction can be useful for the future structural analysis of protein–sugar interactions.

## Methods

### Studied genomes

All studied genomes were downloaded from the MicrobesOnline database [54], which currently contains 17 complete or nearly complete genomes of *Bacteroides* spp.. For comparative analysis, we selected 11 *Bacteroides* genomes: *B. thetaiotaomicron* VPI-5482, *B. ovatus* ATCC 8483, *B. cellulosilyticus* DSM 14838, *B. coprophilus* DSM 18228, *B. dorei* DSM 17855, *B. eggerthii* DSM 20697, *B. fragilis* NCTC 9343, *B. plebeius* DSM 17135, *B. stercoris* ATCC 43183, *B. uniformis* ATCC 8492, and *B. vulgatus* ATCC 8482. We excluded from our analysis the genomes of closely related strains (*B. capillosus* ATCC 29799, *B. coprosuis* DSM 18011, *B. finegoldii* DSM 17565, *B. fragilis* YCH46, *B. helcogenes* P 36-108, and *B. salanitronis* DSM 18170), because they skew the training set of TF-binding sites and thus decrease the sensitivity of the TF-binding site (TFBS) recognition rule.

### Comparative genomics techniques

For *de novo* reconstruction of novel TF regulons, we used two previously developed bioinformatics techniques (reviewed in [27]) that are based on identification and comparative analysis of candidate TFBSs in closely related genomes. The *consistency-check* approach is based on the assumption that regulons (sets of co-regulated genes) have a tendency to be conserved between the genomes that contain orthologous TFs [32,33,55]. The presence of the same TFBS upstream of orthologous genes is an indication that it is a true regulatory site, whereas TFBSs scattered at random in the genome are considered false positives. Simultaneous analysis of multiple genomes from the same taxonomic group allows one to make reliable predictions of TFBSs even with weak recognition rules. The *phylogenetic footprinting* approach is based on the assumption that functional DNA sequences (such as TFBSs) diverge more slowly than nonfunctional ones (e.g., spacers in the intergenic regions) [56,57]. These comparative genomics techniques were previously utilized in several computational workflows for reconstruction of both previously characterized and novel TF regulons in bacteria [20-26].

**Figure 6:**
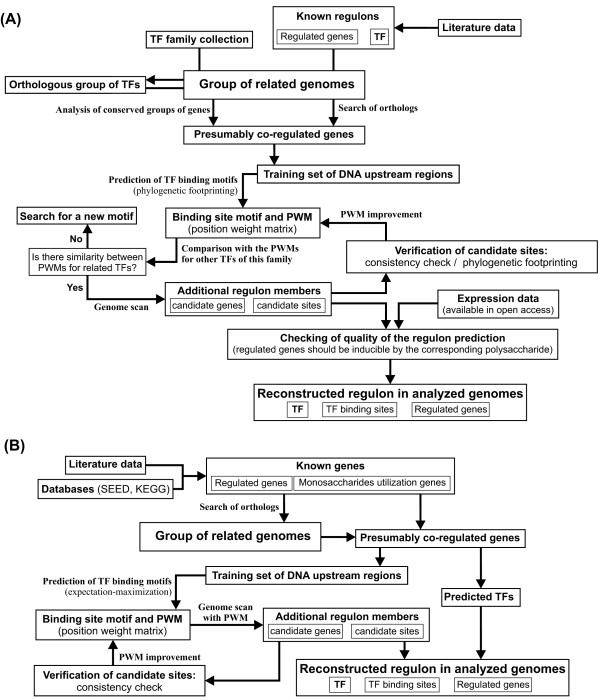
**Bioinformatics workflows used for reconstruction of TF regulons. (A)** Workflow 1 used for identification of DNA binding sites and regulon reconstruction for SusR-like regulators and HTCSs controlling polysaccharide utilization pathways. **(B)** Workflow 2 used for inference of binding site motifs and regulons for TFs controlling monosaccharide utilization pathways.

Below, we describe in more details two computational workflows utilized in this study (Figure 6). Workflow 1 was used for reconstruction of TF regulons operated by the HTCSs and SusR-like regulators, whereas Workflow 2 was used for regulon analysis for TFs belonging to the LacI, GntR, AraC, NrtR, Crp, and ROK families.

### Workflow 1

Workflow 1 is based on the phylogenetic footprinting and comparison of predicted motifs for related TFs (Figure 6A). The following major procedures were performed as a part of Workflow 1: (i) identification of orthologous groups of the studied TFs in *Bacteroides* spp.; (ii) search of genes presumably regulated by a TF; (iii) prediction of candidate TF binding motifs by the phylogenetic footprinting technique and comparison of DNA motifs for related TFs; (iv) construction of motif-specific position weight matrices (PWMs) and genome scan with the constructed PWMs for identification of novel regulon members; (v) checking of quality of the regulon prediction by comparison with the available expression data.

Orthologs of TFs were identified by a procedure based on the analysis of phylogenetic trees for protein domains in MicrobesOnline [54] and validated by bidirectional genome-wide similarity searches with 30% of identity threshold using the Smith-Waterman algorithm implemented in the Genome Explorer program [58]. Multiple alignments of the identified regulators were constructed by MUSCLE [59]. Phylogenetic trees were constructed by the maximum-likelihood method with default parameters implemented in PHYLIP (v. 3.69) [60] and visualized using Dendroscope [61]. Orthologous groups of regulators were identified as high-confidence clusters on the phylogenetic trees constructed for HTCSs and SusR-like systems.

Candidate genes/operons that are potentially regulated by the identified orthologous TFs were predicted by analyzing genomic neighborhoods of each TF gene. We collected the upstream regions of putatively regulated genes from all *Bacteroides* genomes possessing a TF ortholog (from nucleotides −400 to +50 with respect to the translation start). Each set included the TF-encoded operon, as well as one upstream and one downstream operon. We used operon predictions from MicrobesOnline [54].

For the prediction of candidate binding site motifs, we used the phylogenetic footprinting approach that allowed identification of the islands of conservation in the global multiple alignments of orthologous upstream DNA regions [27]. Multiple DNA sequence alignments of orthologous gene upstream regions were constructed by MUSCLE [59]. Also, we utilized an additional criterion of similarity between candidate site motifs for closely related regulators that is based on the expectation that binding sites of regulators with closely related DNA-binding domains are more likely to be at least partially similar [21,31-33]. The predicted TF-binding motifs were compared using Pearson correlation coefficients calculated using Tomtom [62] and further clustered using Hierarchical Clustering Explorer 3.5 [63]. For each novel motif, a specific PWM was constructed using the RegPredict Web server (regpredict.lbl.gov) [64]. Sequence logos for DNA binding motifs were drawn with WebLogo [65].

The constructed PWMs were further used for a whole-genome search of additional candidate binding sites in the *Bacteroides* genomes and their validation via the consistency check approach using the RegPredict Web server. A gene was considered to be a member of a regulon if putative binding sites were found upstream of the gene and upstream of its orthologs in several other genomes bearing orthologous regulators or if it was included in the operon containing such a conserved binding site. Strong non-conserved binding sites were considered true positives if they were identified upstream of genes that are functionally related to known members of the regulon. All identified regulator binding sites were aligned and used for refinement of a binding site motif, building of an updated PWM, and final regulon reconstruction.

### Workflow 2

Workflow 2 is based on the prediction of TFBSs by expectation-maximization techniques. It was used for analysis of regulons for cytoplasmic TFs that belong to well-characterized TF protein families. Workflow 2 includes the following steps: (i) collecting the data about the genes for monosaccharide utilization from literature and database, searching of orthologs of these genes in the analyzed genomes and prediction of the candidate TFs on the basis of genomic context [27]; (ii) prediction of the putative TF binding motifs via analysis of upstream regions of possibly co-regulated genes; (iii) construction of motif-specific PWMs and genome scan for identification of novel regulon members; (v) verification of candidate regulon members by consistency filtering.

As in Workflow 1, we used the same techniques to identify orthologs of the studied TFs, select sets of candidate target operons/genes, and extract their upstream regulatory regions in the studied *Bacteroides* genomes. Then, for each set of upstream DNA sequences, we used the expectation–maximization algorithm implemented in the Discover Profile tool of the RegPredict server [64] to identify a common palindromic motif with the highest information content and construct a PWM. Each TF-specific PWM was further used for a whole genome search using a threshold determined as the smallest score of the site in the training set. The consistency check approach implemented in the RegPredict server [64] was used to predict additional true positive TFBSs and for final regulon reconstruction.

### Other bioinformatics techniques and databases

A detailed (expert) analysis at the last stage of regulon reconstruction includes mechanistic interpretation, prediction of possible effectors of TFs (based on regulated metabolic pathways), and a tentative functional assignment of previously uncharacterized genes. Functional gene annotations were uploaded from SEED [42], UniProt [66], and MicrobesOnline or extracted from the previous metabolic reconstruction of *B. thetaiotaomicron*[10]. Predictions of cellular protein localization were obtained using MetaLocGramN [67].

### Data availability

All predicted regulons including TFs, their binding sites, their regulated genes and operons, and functional gene assignments were deposited in the RegPrecise database [34] and are freely available at: http://regprecise.lbl.gov/RegPrecise/collection_tax.jsp?collection_id=8.

## Abbreviations

TF: Transcription factor; TFBS: Transcription factor-binding site; PUL: Polysaccharide utilization loci; HTCS: Hybrid two-component system; PSU: Polysaccharide and sugar utilization; PWM: Position weight matrix.

## Competing interests

The authors declare that they have no competing interests.

## Authors’ contributions

DARo and ALO conceived and designed the research project. DARa and DARo performed the analysis and wrote the manuscript. AG contributed to the development of the manuscript. All authors read and approved the final manuscript.

## Supplementary Material

Additional file 1 **HTCS and SusR-like proteins identified in *B. thetaiotomicron *** **genomes.** Newly identified regulatory systems are indicated by bold italic font. ^1^” n/s ” is used for regulons that have not been studied in this work; ^2^The presence (+) or absence (−) of SusC/SusD pairs in the cognate PUL; ^3^Experimental data form the literature (for references, see the PMIDs column); ^4^Analysis of the cognate PUL genes functions provided in the current research. Click here for file

Additional file 2 **Multiple alignments of upstream regions for genes regulated by SusR-like proteins, HTCSs and TFs from other protein families.** Binding motifs are shown in red, with the exception of the Crp binding motifs (underlined). Coding regions are in boldface. Genome abbreviations are *Bacteroides thetaiotaomicron* VPI-5482 (BT), *Bacteroides ovatus* ATCC 8483 (BACOVA), *Bacteroides vulgatus* ATCC 8482 (BVU), *Bacteroides dorei* DSM 17855 (BACDOR), *Bacteroides uniformis* ATCC 8492 (BACUNI), *Bacteroides cellulosilyticus* DSM 14838 (BACCELL), *Bacteroides finegoldii* DSM 17565 (BFIN), *Bacteroides faecis* MAJ27 (BFaeM), *Bacteroides* sp. 1_1_14 (1_1_14), *Bacteroides plebeius* DSM 17135 (BACPLE), *Bacteroides dorei* DSM 17855 (BACDOR), *Bacteroides eggerthii* DSM 20697 (BACEGG), *Bacteroides* sp. 1_1_6 (BSIG), *Bacteroides caccae* ATCC 43185 (BACCAC). Click here for file

Additional file 3 **Genes included in the reconstructed regulatory and metabolic networks in *B. thetaiotaomicron. ***^1^Subcellular localization of proteins (E—extracellular, O—outer membrane, P—periplasm, I—inner membrane, C—cytoplasm); ^2^Novel functional roles predicted in this work are shown by light-blue background; ^3^PDB IDs for the proteins with available structure; ^4^Based on the expression data (Martens et al., 2011); ^5^Functional group (E—enzyme, T—transport protein, R—regulatory protein, U—undefined); ^6^Overlap with other models (G—genome-scale reconstruction by Heinken et. al., 2013, M—reconstruction by Model SEED). Click here for file

Additional file 4: Figure S1 Maximum-likelihood phylogenetic tree for HTH domains of the HTCS regulators. Proteins with reconstructed regulons are shown in bold. Click here for file

Additional file 5 **Reconstructed pathways of polysaccharides utilization in *B. thetaiotaomicron.*** Genes in the same regulon and regulators for this regulon are shown by the matching background colors. Click here for file

## References

[B1] Kinross JM Darzi AW Nicholson JK Gut microbiome-host interactions in health and disease Genome Med 2011 14 3 14 21392406 10.1186/gm228 PMC3092099

[B2] Koropatkin NM Cameron EA Martens EC How glycan metabolism shapes the human gut microbiota Nat Rev Microbiol 2012 14 5 323 335 22491358 10.1038/nrmicro2746 PMC4005082

[B3] Round JL Mazmanian SK The gut microbiota shapes intestinal immune responses during health and disease Nat Rev Immunol 2009 14 5 313 323 19343057 10.1038/nri2515 PMC4095778

[B4] Eckburg PB Bik EM Bernstein CN Purdom E Dethlefsen L Sargent M Gill SR Nelson KE Relman DA Diversity of the human intestinal microbial flora Science 2005 14 5728 1635 1638 15831718 10.1126/science.1110591 PMC1395357

[B5] Arumugam M Raes J Pelletier E Le Paslier D Yamada T Mende DR Fernandes GR Tap J Bruls T Batto JM Enterotypes of the human gut microbiome Nature 2011 14 7346 174 180 21508958 10.1038/nature09944 PMC3728647

[B6] Kurokawa K Itoh T Kuwahara T Oshima K Toh H Toyoda A Takami H Morita H Sharma VK Srivastava TP Comparative metagenomics revealed commonly enriched gene sets in human gut microbiomes DNA Res 2007 14 4 169 181 17916580 10.1093/dnares/dsm018 PMC2533590

[B7] Hooper LV Midtvedt T Gordon JI How host-microbial interactions shape the nutrient environment of the mammalian intestine Annu Rev Nutr 2002 14 283 307 12055347 10.1146/annurev.nutr.22.011602.092259

[B8] Xu J Bjursell MK Himrod J Deng S Carmichael LK Chiang HC Hooper LV Gordon JI A genomic view of the human-*Bacteroides thetaiotaomicron* symbiosis Science 2003 14 5615 2074 2076 12663928 10.1126/science.1080029

[B9] Sonnenburg ED Zheng H Joglekar P Higginbottom SK Firbank SJ Bolam DN Sonnenburg JL Specificity of polysaccharide use in intestinal bacteroides species determines diet-induced microbiota alterations Cell 2010 14 7 1241 1252 20603004 10.1016/j.cell.2010.05.005 PMC2900928

[B10] Heinken A Sahoo S Fleming RM Thiele I Systems-level characterization of a host-microbe metabolic symbiosis in the mammalian gut Gut Microbes 2013 14 1 28 40 23022739 10.4161/gmic.22370 PMC3555882

[B11] Cantarel BL Coutinho PM Rancurel C Bernard T Lombard V Henrissat B The Carbohydrate-Active EnZymes database (CAZy): an expert resource for Glycogenomics Nucleic Acids Res 2009 14 Database issue D233 D238 18838391 10.1093/nar/gkn663 PMC2686590

[B12] D’Elia JN Salyers AA Effect of regulatory protein levels on utilization of starch by *Bacteroides thetaiotaomicron* J Bacteriol 1996 14 24 7180 7186 8955400 10.1128/jb.178.24.7180-7186.1996 PMC178631

[B13] Bolam DN Koropatkin NM Glycan recognition by the bacteroidetes Sus-like systems Curr Opin Struct Biol 2012 14 5 563 569 22819666 10.1016/j.sbi.2012.06.006

[B14] Martens EC Roth R Heuser JE Gordon JI Coordinate regulation of glycan degradation and polysaccharide capsule biosynthesis by a prominent human gut symbiont J Biol Chem 2009 14 27 18445 18457 19403529 10.1074/jbc.M109.008094 PMC2709373

[B15] Lowe EC Basle A Czjzek M Firbank SJ Bolam DN A scissor blade-like closing mechanism implicated in transmembrane signaling in a *Bacteroides* hybrid two-component system Proc Natl Acad Sci USA 2012 14 19 7298 7303 22532667 10.1073/pnas.1200479109 PMC3358863

[B16] Martens EC Koropatkin NM Smith TJ Gordon JI Complex glycan catabolism by the human gut microbiota: the *Bacteroidetes* Sus-like paradigm J Biol Chem 2009 14 37 24673 24677 19553672 10.1074/jbc.R109.022848 PMC2757170

[B17] Xu J Chiang HC Bjursell MK Gordon JI Message from a human gut symbiont: sensitivity is a prerequisite for sharing Trends Microbiol 2004 14 1 21 28 14700548 10.1016/j.tim.2003.11.007

[B18] Martens EC Chiang HC Gordon JI Mucosal glycan foraging enhances fitness and transmission of a saccharolytic human gut bacterial symbiont Cell Host Microbe 2008 14 5 447 457 18996345 10.1016/j.chom.2008.09.007 PMC2605320

[B19] Fischbach MA Sonnenburg JL Eating for two: how metabolism establishes interspecies interactions in the gut Cell Host Microbe 2011 14 4 336 347 22018234 10.1016/j.chom.2011.10.002 PMC3225337

[B20] Leyn SA Kazanov MD Sernova NV Ermakova EO Novichkov PS Rodionov DA Genomic reconstruction of the transcriptional regulatory network in *Bacillus subtilis* J Bacteriol 2013 14 11 2463 2473 23504016 10.1128/JB.00140-13 PMC3676070

[B21] Ravcheev DA Best AA Sernova NV Kazanov MD Novichkov PS Rodionov DA Genomic reconstruction of transcriptional regulatory networks in lactic acid bacteria BMC Genomics 2013 14 1 94 23398941 10.1186/1471-2164-14-94 PMC3616900

[B22] Ravcheev DA Best AA Tintle N Dejongh M Osterman AL Novichkov PS Rodionov DA Inference of the transcriptional regulatory network in *Staphylococcus aureus* by integration of experimental and genomics-based evidence J Bacteriol 2011 14 13 3228 3240 21531804 10.1128/JB.00350-11 PMC3133287

[B23] Rodionov DA Novichkov PS Stavrovskaya ED Rodionova IA Li X Kazanov MD Ravcheev DA Gerasimova AV Kazakov AE Kovaleva GY Comparative genomic reconstruction of transcriptional networks controlling central metabolism in the *Shewanella* genus BMC Genomics 2011 14 Suppl 1 S3 21810205 10.1186/1471-2164-12-S1-S3 PMC3223726

[B24] Rodionov DA Rodionova IA Li X Ravcheev DA Tarasova Y Portnoy VA Zengler K Osterman AL Transcriptional regulation of the carbohydrate utilization network in *Thermotoga maritima* Front Microbiol 2013 14 244 23986752 10.3389/fmicb.2013.00244 PMC3750489

[B25] Rodionov DA Li X Rodionova IA Yang C Sorci L Dervyn E Martynowski D Zhang H Gelfand MS Osterman AL Transcriptional regulation of NAD metabolism in bacteria: genomic reconstruction of NiaR (YrxA) regulon Nucleic Acids Res 2008 14 6 2032 2046 18276644 10.1093/nar/gkn046 PMC2330245

[B26] Rodionov DA Mironov AA Rakhmaninova AB Gelfand MS Transcriptional regulation of transport and utilization systems for hexuronides, hexuronates and hexonates in gamma purple bacteria Mol Microbiol 2000 14 4 673 683 11115104 10.1046/j.1365-2958.2000.02115.x

[B27] Rodionov DA Comparative genomic reconstruction of transcriptional regulatory networks in bacteria Chem Rev 2007 14 8 3467 3497 17636889 10.1021/cr068309+ PMC2643304

[B28] Martens EC Lowe EC Chiang H Pudlo NA Wu M McNulty NP Abbott DW Henrissat B Gilbert HJ Bolam DN Recognition and degradation of plant cell wall polysaccharides by two human gut symbionts PLoS Biol 2011 14 12 e1001221 22205877 10.1371/journal.pbio.1001221 PMC3243724

[B29] Rogers TE Pudlo NA Koropatkin NM Bell JS Moya Balasch M Jasker K Martens EC Dynamic responses of Bacteroides thetaiotaomicron during growth on glycan mixtures Mol Microbiol 2013 14 5 876 890 23646867 10.1111/mmi.12228 PMC3700664

[B30] Sonnenburg ED Sonnenburg JL Manchester JK Hansen EE Chiang HC Gordon JI A hybrid two-component system protein of a prominent human gut symbiont couples glycan sensing in vivo to carbohydrate metabolism Proc Natl Acad Sci USA 2006 14 23 8834 8839 16735464 10.1073/pnas.0603249103 PMC1472243

[B31] Kazanov MD Li X Gelfand MS Osterman AL Rodionov DA Functional diversification of ROK-family transcriptional regulators of sugar catabolism in the Thermotogae phylum Nucleic Acids Res 2013 14 2 790 803 23209028 10.1093/nar/gks1184 PMC3553997

[B32] Leyn SA Li X Zheng Q Novichkov PS Reed S Romine MF Fredrickson JK Yang C Osterman AL Rodionov DA Control of Proteobacterial central carbon metabolism by the HexR transcriptional regulator: a case study in *Shewanella oneidensis* J Biol Chem 2011 14 41 35782 35794 21849503 10.1074/jbc.M111.267963 PMC3195618

[B33] Ravcheev DA Li X Latif H Zengler K Leyn SA Korostelev YD Kazakov AE Novichkov PS Osterman AL Rodionov DA Transcriptional regulation of central carbon and energy metabolism in bacteria by redox responsive repressor Rex J Bacteriol 2012 14 5 1145 1157 22210771 10.1128/JB.06412-11 PMC3294762

[B34] Novichkov PS Kazakov AE Ravcheev DA Leyn SA Kovaleva GY Sutormin RA Kazanov MD Riehl W Arkin AP Dubchak I RegPrecise 3.0–a resource for genome-scale exploration of transcriptional regulation in Bacteria BMC Genomics 2013 14 745 24175918 10.1186/1471-2164-14-745 PMC3840689

[B35] Xu J Mahowald MA Ley RE Lozupone CA Hamady M Martens EC Henrissat B Coutinho PM Minx P Latreille P Evolution of symbiotic bacteria in the distal human intestine PLoS Biol 2007 14 7 e156 17579514 10.1371/journal.pbio.0050156 PMC1892571

[B36] Kleene R Schachner M Glycans and neural cell interactions Nat Rev Neurosci 2004 14 3 195 208 14976519 10.1038/nrn1349

[B37] Abbott DW Boraston AB Structural biology of pectin degradation by Enterobacteriaceae Microbiol Mol Biol Rev 2008 14 2 301 316 table of contents 18535148 10.1128/MMBR.00038-07 PMC2415742

[B38] Willats WG McCartney L Mackie W Knox JP Pectin: cell biology and prospects for functional analysis Plant Mol Biol 2001 14 1-2 9 27 11554482

[B39] Patel EH Paul LV Casanueva AI Patrick S Abratt VR Overexpression of the rhamnose catabolism regulatory protein, RhaR: a novel mechanism for metronidazole resistance in *Bacteroides thetaiotaomicron* J Antimicrob Chemother 2009 14 2 267 273 19525515 10.1093/jac/dkp203 PMC2707267

[B40] Patel EH Paul LV Patrick S Abratt VR Rhamnose catabolism in *Bacteroides thetaiotaomicron* is controlled by the positive transcriptional regulator RhaR Res Microbiol 2008 14 9-10 678 684 18848625 10.1016/j.resmic.2008.09.002

[B41] Hooper LV Xu J Falk PG Midtvedt T Gordon JI A molecular sensor that allows a gut commensal to control its nutrient foundation in a competitive ecosystem Proc Natl Acad Sci USA 1999 14 17 9833 9838 10449780 10.1073/pnas.96.17.9833 PMC22296

[B42] Disz T Akhter S Cuevas D Olson R Overbeek R Vonstein V Stevens R Edwards RA Accessing the SEED genome databases via Web services API: tools for programmers BMC Bioinforma 2010 14 319 10.1186/1471-2105-11-319 PMC2900279 20546611

[B43] Overbeek R Begley T Butler RM Choudhuri JV Chuang HY Cohoon M de Crecy-Lagard V Diaz N Disz T Edwards R The subsystems approach to genome annotation and its use in the project to annotate 1000 genomes Nucleic Acids Res 2005 14 17 5691 5702 16214803 10.1093/nar/gki866 PMC1251668

[B44] Kanehisa M Goto S Hattori M Aoki-Kinoshita KF Itoh M Kawashima S Katayama T Araki M Hirakawa M From genomics to chemical genomics: new developments in KEGG Nucleic Acids Res 2006 14 D354 D357 16381885 10.1093/nar/gkj102 PMC1347464

[B45] Kanehisa M Goto S KEGG: kyoto encyclopedia of genes and genomes Nucleic Acids Res 2000 14 1 27 30 10592173 10.1093/nar/28.1.27 PMC102409

[B46] Conejo MS Thompson SM Miller BG Evolutionary bases of carbohydrate recognition and substrate discrimination in the ROK protein family J Mol Evol 2010 14 6 545 556 20512568 10.1007/s00239-010-9351-1

[B47] Hoskisson PA Rigali S Chapter 1: variation in form and function the helix-turn-helix regulators of the GntR superfamily Adv Appl Microbiol 2009 14 1 22 19729089 10.1016/S0065-2164(09)69001-8

[B48] Swint-Kruse L Matthews KS Allostery in the LacI/GalR family: variations on a theme Curr Opin Microbiol 2009 14 2 129 137 19269243 10.1016/j.mib.2009.01.009 PMC2688824

[B49] Yang J Tauschek M Robins-Browne RM Control of bacterial virulence by AraC-like regulators that respond to chemical signals Trends Microbiol 2011 14 3 128 135 21215638 10.1016/j.tim.2010.12.001

[B50] Rodionov DA De Ingeniis J Mancini C Cimadamore F Zhang H Osterman AL Raffaelli N Transcriptional regulation of NAD metabolism in bacteria: NrtR family of Nudix-related regulators Nucleic Acids Res 2008 14 6 2047 2059 18276643 10.1093/nar/gkn047 PMC2330246

[B51] Teramoto H Suda M Inui M Yukawa H Regulation of the expression of genes involved in NAD de novo biosynthesis in *Corynebacterium glutamicum* Appl Environ Microbiol 2010 14 16 5488 5495 20601509 10.1128/AEM.00906-10 PMC2918977

[B52] Grainger DC Hurd D Harrison M Holdstock J Busby SJ Studies of the distribution of Escherichia coli cAMP-receptor protein and RNA polymerase along the E. coli chromosome Proc Natl Acad Sci USA 2005 14 49 17693 17698 16301522 10.1073/pnas.0506687102 PMC1308901

[B53] Devoid S Overbeek R DeJongh M Vonstein V Best AA Henry C Automated genome annotation and metabolic model reconstruction in the SEED and Model SEED Methods Mol Biol 2013 14 17 45 PubMed PMID: 23417797 23417797 10.1007/978-1-62703-299-5_2

[B54] Dehal PS Joachimiak MP Price MN Bates JT Baumohl JK Chivian D Friedland GD Huang KH Keller K Novichkov PS MicrobesOnline: an integrated portal for comparative and functional genomics Nucleic Acids Res 2010 14 D396 D400 19906701 10.1093/nar/gkp919 PMC2808868

[B55] Kazakov AE Rodionov DA Alm E Arkin AP Dubchak I Gelfand MS Comparative genomics of regulation of fatty acid and branched-chain amino acid utilization in proteobacteria J Bacteriol 2009 14 1 52 64 18820024 10.1128/JB.01175-08 PMC2612455

[B56] McCue L Thompson W Carmack C Ryan MP Liu JS Derbyshire V Lawrence CE Phylogenetic footprinting of transcription factor binding sites in proteobacterial genomes Nucleic Acids Res 2001 14 3 774 782 11160901 10.1093/nar/29.3.774 PMC30389

[B57] Shelton DA Stegman L Hardison R Miller W Bock JH Slightom JL Goodman M Gumucio DL Phylogenetic footprinting of hypersensitive site 3 of the beta-globin locus control region Blood 1997 14 9 3457 3469 9129054

[B58] Mironov AA Vinokurova NP Gel’fand MS Software for analyzing bacterial genomes Mol Biol (Mosk) 2000 14 2 253 262 10779952

[B59] Edgar RC MUSCLE: multiple sequence alignment with high accuracy and high throughput Nucleic Acids Res 2004 14 5 1792 1797 15034147 10.1093/nar/gkh340 PMC390337

[B60] Felsenstein J Inferring phylogenies from protein sequences by parsimony, distance, and likelihood methods Meth Enzymol 1996 14 418 427 8743697 10.1016/s0076-6879(96)66026-1

[B61] Huson DH Richter DC Rausch C Dezulian T Franz M Rupp R Dendroscope: an interactive viewer for large phylogenetic trees BMC Bioinforma 2007 14 460 10.1186/1471-2105-8-460 PMC2216043 18034891

[B62] Gupta S Stamatoyannopoulos JA Bailey TL Noble WS Quantifying similarity between motifs Genome Biol 2007 14 2 R24 17324271 10.1186/gb-2007-8-2-r24 PMC1852410

[B63] Seo J Gordish-Dressman H Exploratory data analysis with categorical variables: an improved rank-by-feature framework and a case study Int J Hum-Comp Interact 2007 14 3 287 314

[B64] Novichkov PS Rodionov DA Stavrovskaya ED Novichkova ES Kazakov AE Gelfand MS Arkin AP Mironov AA Dubchak I RegPredict: an integrated system for regulon inference in prokaryotes by comparative genomics approach Nucleic Acids Res 2010 14 W299 W307 20542910 10.1093/nar/gkq531 PMC2896116

[B65] Crooks GE Hon G Chandonia JM Brenner SE WebLogo: a sequence logo generator Genome Res 2004 14 6 1188 1190 15173120 10.1101/gr.849004 PMC419797

[B66] Magrane M Consortium U UniProt knowledgebase: a hub of integrated protein data Database (Oxford) 2011 14 bar009 21447597 10.1093/database/bar009 PMC3070428

[B67] Magnus M Pawlowski M Bujnicki JM MetaLocGramN: a meta-predictor of protein subcellular localization for Gram-negative bacteria Biochim Biophys Acta 2012 14 12 1425 1433 22705560 10.1016/j.bbapap.2012.05.018

