# Supplementary material for: Polysaccharides utilization in human gut bacterium Bacteroides thetaiotaomicron: comparative genomics reconstruction of metabolic and regulatory networks
Source: BMC Genomics. 2013 Dec 12;14:873. doi: 10.1186/1471-2164-14-873 (PMC3878776; doi:10.1186/1471-2164-14-873)
Supplement: Additional file 2 — Multiple alignments of upstream regions for genes regulated by SusR-like proteins, HTCSs and TFs from other protein families. Binding motifs are shown in red, with the exception of the Crp binding motifs (underlined). Coding regions are in boldface. Genome abbreviations are Bacteroides thetaiotaomicron VPI-5482 (BT), Bacteroides ovatus ATCC 8483 (BACOVA), Bacteroides vulgatus ATCC 8482 (BVU), Bacteroides dorei DSM 17855 (BACDOR), Bacteroides uniformis ATCC 8492 (BACUNI), Bacteroides cellulosilyticus DSM 14838 (BACCELL), Bacteroides finegoldii DSM 17565 (BFIN), Bacteroides faecis MAJ27 (BFaeM), Bacteroides sp. 1_1_14 (1_1_14), Bacteroides plebeius DSM 17135 (BACPLE), Bacteroides dorei DSM 17855 (BACDOR), Bacteroides eggerthii DSM 20697 (BACEGG), Bacteroides sp. 1_1_6 (BSIG), Bacteroides caccae ATCC 43185 (BACCAC). [file 1471-2164-14-873-S2.pdf]

Multiple alignments of upstream regions for genes regulated by SusR-like proteins, HTCS and cytoplasmic transcriptional regulators.

Binding motifs are shown in red, with the exception of the Crp binding motifs (underlined). Coding regions are in boldface. Genome abbreviations are *Bacteroides thetaiotaomicron* VPI-5482 (BT), *Bacteroides ovatus* ATCC 8483 (BACOVA), *Bacteroides vulgatus* ATCC 8482 (BVU), *Bacteroides dorei* DSM 17855 (BACDOR), *Bacteroides uniformis* ATCC 8492 (BACUNI), *Bacteroides cellulosilyticus* DSM 14838 (BACCELL), *Bacteroides finegoldii* DSM 17565 (BFIN), *Bacteroides faecis* MAJ27 (BFaeM), *Bacteroides* sp. 1_1_14 (1_1_14), *Bacteroides plebeius* DSM 17135 (BACPLE), *Bacteroides dorei* DSM 17855 (BACDOR), *Bacteroides eggerthii* DSM 20697 (BACEGG), *Bacteroides* sp. 1_1_6 (BSIG), *Bacteroides caccae* ATCC 43185 (BACCAC).

SusR (BT3705) regulon

```
BVU_1382          ACGAAAATATTTTCATTGACGTTTATATTTTCAGCAATACAGCAAAGACAAATATTCCTAATTATCAATAAGTTGAGTCTAT
BACDOR_02286      ACGAAAATATTTTCATTGACGTTTATATTTTCAGCAATACAGCAGAGATAAATACACCTAGTTATCAATAAGTTGAGTCTAT
BT3704            CCATAACAATTTTCATTTTCACTTGTATTTTTCGACAGAGGCAATAACGATAAAAAGCTTATTATCAACAGCCTATTCAAAA
BACOVA_03520      GTATAACTATTTTCATTTTCACTTGTATTTTTCGGCATAAGGTATTAGAATATCCAACTAATTATCAGCAACATAGTCAGAT
                      **  *********  *  ** ******  *  * *    *     * *    **  ******  *   *     *

BVU_1382          AAAACATTTATATTTTTATTCTAGTACTCAAATCTCAGCTATTCCACTCCTACATTTGCAACATCCA--AATGAAATGAAGA
BACDOR_02286      AAAACATTTATATTTTTATTCTAGTACTCAAATCTCATCTATTCCACTCCTACATTTGCAACATCCA--AATGAAATGAAGA
BT3704            CAAGCATTTATATTTTCTTTCTAACTGCCATACGGCATACCCGATTCACCTACATTTGCATCATCGGTTCCCCCGAACAAAT
BACOVA_03520      AACAGGTTTATATTTCCAATTCTAATCTCACAAAGGTGCACAGCATCCCCCTACATTTGCATCATCGGTTCTCCGAGCAAACA
                   *    *********   *****        *             * ************ ****            **

BVU_1382          TATAAACACTAATTTCTATATATTATGAAAAGAA------CATTCGCACTTA
BACDOR_02286      TATAAACACTAATTTCTATATATTATGAAAAGAA------CATTCGCACTTA
BT3704            TTTAAAAA------TCTAGTTACCATGAAAAGGAATTTATTATTCATTATCT
BACOVA_03520      GTTAAAAA------TCTAGCTACCATGAAACGAAATTTGTTATTTGCTATTT
                    **** *      **  **  **  ****** * *      ***     *
```

SusR2 (BT3091) regulon

```
BT3090             ---AGAAATCCACTACTTTTTTAGCACGGTCGCTATTCTATAATATTCTTATTATTAGTGTTTTATGCTGTTTTGTACTGT
BACOVA_02787       ---AGAAAATCACTACTTTTTTAGTCTGACTTCTATTTTATAACGTTCTTATATTTAGTGGTTTATGCTGTGAGATACTGT
BACUNI_01942       CTTATATTTTCACTACTTTTTTAGTTGGGTAATAATTTTATATATTATTGGTATATAGTGGTTTAAGTATTTTTGAATGAT
BACCELL_04952      CTTATATTTTTACTACTTTTTTGGAACAATGCATCTTCTTGATAGCATTGATTATTAGTGTTTTATGTCTTATAAAACGCC
                      * *    *********** *        ** *  *     *  *  ***** **** *   *    *

BT3090             ATAAACCACTATATTTTTGCTATCTGGCAAAGAAGCGCATTTAATTGTGGATACTTTTGAT----------TTCAG-----
BACOVA_02787       ATAAATCACTATATTTTTGCTATCTGCTTAAGAAGCAGTATGAAATGTGGATACTTTTGTTTTCACGGTCATTCAGCAATG
BACUNI_01942       GTAAACCACTACTTTTTTACTCTTTCCTCACTATTGGCTTCTCTTATCTGATACATTTGCCATTGTCCCTCTTCGG-----
BACCELL_04952      TAAAACTACTACTTTTTCACTCTTTTCCCCTTATTGCTTCCTCTTATCAGATACATTTGCCATTGTTCCTTCACAG-----
                     ***  ****  ****  ** * *      *              ***** ****           * *

BT3090             ----TGAAAACAAAA-----------AGTAATCCTAATATTAACTTTAAATTGATGTA-CATGGAACAGAGTATAAA
BACOVA_02787       ACACAGAGAACGAAAACAAA------AGTAACCCTAATATTAACTTTAAATTAATGTA-CATGAAACAGAGTATGAA
BACUNI_01942       ----AAAGAGTGACA-CATGTGATT-TATGTTTTTATTATTAACTTTAAATTCTTATATTATGCAAAAGTACAAAAT
BACCELL_04952      ----AGGGGACAAATGCATGTGAATCTATATTTTTATTACTAACTTTAAACTCTTATATTATGCAAAAGTACAAAAT
                              *             *    ** ** ********** *  * **  *** ** **   *  *
```

SusR3 (BT3309) regulon

```
BT3310            TCGATGTTGTAACCTATCCACTTTTTAGGCCTCTTTGGTTGATATTAACTTGCTGTTTATTAGTGGTTTGTATGTTTGTAT
BACOVA_00942      TTAAGACTGTAAACTAACCACTTTTTTGGATATGTTATATGTGTGTAAATGGTTGATAATCATTTTGATGATGTTTTGTGT
BACFIN_00069      TCAAGGTTGTAAACTATCCACTTTTTTAGACTGAAGAAAAGAGAGTAAGCTTTTGGAAATGAATTGTTTGCCTTTTAATGG
                  *  *   ***** *** *********  *         *    ***     **    ** * *    **    **  *

BT3310            AATCCACTTATTATACCCTCATTGTCAAATCACTTCAGTTTCTTTATACTTTTGACCTGTGCTTCTGAAATAGTGGCGAAA
BACOVA_00942      CATCCACTTATTGACCCCTCCCTGTTAGCTCACTTTAATTTCTTTATACTTTTGATCTGTGCTTCGAAGGTAATGAGAACA
BACFIN_00069      TATCCACTTATTGTCCCCTCCTTATGAATTCGTTCTCATTTCTTTATAATTTTGACCTGTGCTTCGGAAGCAATGAGAGGA
                   ***********   *****  * * *  **  *    ********** ****** *********  *   * **     *

BT3310            GTCCTGTCAAGCACATACCTTAATTATAAACTTTAAAA--TTAATACGCATGAAAAAG
BACOVA_00942      G--CTATTACGGACGAGCATCTATTATTAACTTTAAAATTTTAGTACGCATGAAAAAG
BACFIN_00069      ACTCCATTGCGGACGGACGTTTATTATTAACTTTAAAA--TTAGTACGCATGAAAAAG
                     *  *   * **   * *  ***** **********  *** **************
```

SusR4 (BT2160) regulon

```
BACOVA_03187     ATAATACTTTCCAAATCATCTACATTTTTTTGATGTGTCAAGTTAGCTAAGTTGTTGATTAATAGTGAAAAATGCTATTTT
BT2159           AAAATACTTTCCAAACAATCTACATTTTTTATATCCTTGTGTATATCTAAAGTGTTGATTTATAATATGTAATATGATTTT
BFaeM_23198      AAAGCCCTTTCCAAACTGTCTACATTTTTTAGATAGGTAAATATAGATAAGTGTTTGATATATAGAGGTAAATGTGTTTTT
                 * *   *********   ************  **   *     **  ***    *****  ***      ***    ****

BACOVA_03187     TATATCTACATAAAGTAGTGTAAGTCAAATAAGCCGTTATTTATTTGGCTAGATTTGCAATATCAGTTTTTAGCTCATCAG
BT2159           ATAATCTACATAAAGTTGTGTAGACTAAATAAAGCGTTATTTATTTGGCTAGTTTTGCGATATCAGTTTTCGAACAATCTG
BFaeM_23198      TGAATCTACATAAAGTTGTGTAGACTCCATAAAGCGTTATTTATTTGGCTAGTTTTGCGATATCGGTTTTCAGACAATTTG
                    ************* *****      ****  ****************** ***** ***** *****      **  *

BACOVA_03187     TTTGGTAGTTAGAAGTTGATGTTTTATCTATTATTTGTACTTAAATTCACTATGTTTAATTATGAACACGCAATCTTCA
BT2159           CCT-ACAGATGAAAATTGATACCTTTTATGTTGTTTGTACTTAAATTCGCAATTTT---TTATGGATGCACAATTTTCA
BFaeM_23198      CGA-ACGGATGAAAATCGGTATCTTATATATTGTTTGTACTTAAATTCGCCATGTT---TTATGAATACACAATTTTCA
                        * *  ** * * *   ** * * ** *************** * ** **   ***** *  * **** ****
```

HTCS_Aga-1 (BT0267) regulon

```
BACFIN_07018     CAATTATGATTCAAAATTGTACGTTTCTGAATGATTATAGGACTTCTTTTTATTGGATAACTCGCACTTTTGTACTGCAAT
BfaeM_12438      TAATTATGGTCCAATTTTGGAAGTTTTTGAATGATTAGAGAACTTCTTTCTACTGGATAACTCGCACTTTTGTAACGCATT
BT0268           CGATTATGGTCCAATTTTGGAAGTTTTTGAATGATTAGAGAACTTCTTTCTACTGGATAACTCGCACTTTTGTGACGCATT
1_1_14_01201     CGATTATGGTCCAATTTTGGAAGTTTTTGAATGATTAGAGAACTTCTTTCTACTGGATAACTCGCACTTTTGTGATGCATT
                   ****** * ***  *** * **** ********** ** ******** ** ********************   *** *

BACFIN_07018     TAATGCATAACTAATACTTATATGATCTAAAAAAC-TTATAAATCTAATAGCATGGTGGTA
BfaeM_12438      TAATGCATAACTAATACTTA--TGATCTAAATAAC-TCATAAATCTAATCGTATGGTGATA
BT0268           TGATGCACAACTAATACTTATTTGGTCTAAATAACTTTATAAATCTAATAGTATGGTGATA
1_1_14_01201     TGATGCACAACTAATACTTATTTGGTCTAAATAACTTTATAAATCTAATAGTATGGTGATA
                 * ***** ************  ** ****** *** * *********** * ****** **
```

HTCS_Ara-1 (BT0366) regulon

```
BACCELL_05304    AATCAGAATAGCTGTTTTTAATTTAAATTGCTGAATTGTCCACCTCAAATGTTCATTTGTCCACCTTACCATGCTTACCCC
BACEGG_01542     AAAAAGTCTGCGGTCTTTTTTGTGAAGTTGGAAGATTGTCCACCTTAAAAGGTCATATATCCACCTCTACTGCTTTGGGCA
BT0367           AATGGGAAGAGCTTCTTTTCAGC-AGATTATCGGATTGTCCACCAATCGTGTTCGTATGTCCACCTTTCATAGGGGAAACG
                 **   *         ****     *  **     **********      * ** * * *******             *

BACCELL_05304    GGGCGTATTTTTGCAATAGAATTTAT-----AATCTACTGATAGTATG
BACEGG_01542     TGGCGTACATTTGCATT-GTATTTTT-----AATTTGCTGATATTATG
BT0367           GGGCGTATTTTTGTAGCATAATTGATGAACGAATACACAGATATTATG
                  ******  **** *     ***  *     ***   * **** ****
```

HTCS_Ara-2 (BT3049) regulon

```
BACUNI_03755     TCAACAGCTGTATAAATCTTGCAGAGAAGGCATATAAATCTGTCAATGCACGATTTGTGCACATCATCTAAAGATTTTATT
BACCELL_01904    TCTTGAACTGTATAAATCTTGCAGTGAGGGGATGTAAATCTTGCAATGAACGATTTGTGTATCTGAAATAAAGAATTTATT
BVU_0839         TCCTGA----TATAAATCTTGCAGAAAAGGGAGGTAAATCTTGCAATGCATGA-TTATGTATCTAAAATACAGACTTTATT
BACDOR_04890     TCCTGA----TATAAATCTTGCAGAAAAGGGAGGTAAATCTTGCAATGCATGATTTATGTATCTAAAATACAGACTTTATT
BACPLE_00182     TAAGAA----AAAACATCATGCAATGACCGAGCACAAATCATTCATTGTATGATTTACATCCCTTTAATTCAATAAATATT
BT3049           CGCTG-----TAAAAATCATGCAGAAAGGGTAGA-AAATCATGCAATGAATCATTTGTCTATACTAAATACAAGATTTATT
BACOVA_02667     TGAATAAA--CAAAAATCATGCAGAAGAACTACACAAATCATGCAATGAACGATTTGTATACTCTAAATAAAATATTTATT
BACFIN_04000     AACCGA----TAAAAATCATGCAGAGAAGCTACATAAATCATGCAATGCACGATTTACATACTCTAAATAAAATATTTATT
                            * * *** ****            *****   ** ** *  * **            *  *     ****

BACUNI_03755     TGTACTTTTGA----------CTCATGCAATGAAAAACGACCCGTTATGAAGA-AGATTCT
BACCELL_01904    TATACTTTTGGATTGCAATA-----------GAAAATTGAT--GTCATGAAAA-GCAGATT
BVU_0839         TGTATTTTTGAACCATATTATTTAACAATGTGTACACTGAT--GCTATGAGAA-AGATATT
BACDOR_04890     TGTATTTTTGAACCATATTATTTAACAATGTGTACACTGAT--GCTATGAGAA-AGATATT
BACPLE_00182     ACTATTTTTGA--------ATCTACCAAAATGAACAA--------TATGAA---ATATCTT
BT3049           TGTACTTTTGAGAGTGAATATCAAGGAATACATAATATCTGAAACGATGAAAACATACATT
BACOVA_02667     CCTATTTTTGGACTTG-----TAATTGAAACAAATAAGCACGAGTTATGAAAAAACACATT
BACFIN_04000     TCTATTTTTGAGCCTGTAAA-----TGAAACGAATAGGCACGAGTTATGAAAAGACACATT
                   ** *****                       *            ****      *   *
```

HTCS_Fru (BT1754) regulon

```
BT1763          CTATGCAACATATCTTACAGCATTTGAAACATTTTTTTCAGTCGGTT--GACTTAAATCAACCGTAATTTG
BACSTE_03472    CCACGAAACAAATTTTGCAGCATCCGTATCATTTTTTATAGCCAGTTTCAATGCAATCC--ATTTACTTTG
BACCELL_05510   TTTTGTTACAAATATACCAGCTTATGAAACATAAATTACAGCATATTTTCACTTAATTC--CCTTTCTTTG
                    *  *** ** *  **** *  * * ***   **  **    **   *   **  *     *  ****

BT1763          CATCATCGAAAAGAGA-----AAATTCGTACTTTTA-----AATTAATAACATTAAACTAATGCCTGGTA-
BACSTE_03472    CAATATCAAAACATGA-TCGGGAAAGC-TGTTTTGAG----AATTCACAAGA-------------------
BACCELL_05510   CAACATCAAAACATCACTCGGGAAAGCATGTTTTGAGAGGTAATCCACAGCGATTACAAAATGAAAAGCAT
                **  *** ***    *      **  * *  *** *     ***  * *
```

HTCS_Hep (BT4663) regulon

1)

```
BACSTE_03702    TTTTTAAACAAATACGATATTTCATTCAACAAATTTAATAAT-AAAATTCAGATTTCAAAGAAAGCATAAACAAGAGAGAT
BACOVA_05478    TTTCTAAACATTATTAATATATGATTCAACAATATTAATATCAAAAAATCATTTTTTGAACAAAGTGTGAACAGGACAAAT
BT4662          TTTTTAAACAAAAACAACATATGTCTCAACAAATTTGATATCAAAAAAATCATTTTTGAACAAAGTATGAACAACCTAAAT
BACFIN_00658    CTTTTGAACATAAATAGTATATGATTCAACAAAACTAATATCAAAAAAACA-TTTTTGAACAAAGCCTGAACAACTTCGGC
                 ** * ****        ** *   *******   * ***   ****      ***  ** ****  * ****

BACSTE_03702    TAGCTCTTAGAAATCAATGTAATATATTTGCGGCAAGGAAAATTGAAACTTTTACTAAT--AAAAAACAACTTAAGTATGA
BACOVA_05478    CATCATTTCTAAATAAACAGATTACATTTGCAAACAGTAAAA-TATAACTTTTAATAAT--TAAAACCTAATAAAGTATGA
BT4662          TAAAGAATCTAATTAATGAGGTTACATTTGCAA-CAGTAAAA-TAACACTTTTAATAATTATAAAACCTAATAAAGTATGA
BACFIN_00658    CAATCGTTATATATAAAGAGATTACATTTGCAAACAGTAAAA-TATAACTTTTAATAAT--TAAAACCTAATAAAGTATGA
                 *     *  *  * *      ** ******    ** **** *   ******* ****   **** * * * ********
```

2)

```
BACEGG_00070    TTAGGATAGTGACGAATTGCACAAATATAGGGTTTTCTGAAACAAATAGGATAGGGGC-TGAACAATAGCTTTCGTATTTT
BACSTE_03699    GTGGAG-------TATTTGCACAAATATAGGGTTTTCCGAAACAAATAGGGGATAGGTGTGAGGCGCAGCTTTCGTACTTT
BT4675          ACAGAA-------GAATTGCACAAATGTGAAAGCATTTGAGACAAACGAAAGGCAGCCGTAAGGGTTGCCTTTCGTATTTT
BACOVA_05497    ACAGTA-------AAATTACACAAATGTGAAAACATTTGAGACAAAAGAAAGGCAGCCGATGAGGTTGCCTTTCGTATTTT
                   *          * ** ******* *       *  ** *****         *             ******** ***

BACEGG_00070    TGTGACATAA-ATTTAAGCTTAATATCAAAATAGGATGAAGAAAAACATTTT
BACSTE_03699    TGCCGCATCGTTTTTAAGTTTAATATCAAATAAGGATGAAGAAAAACATTTT
BT4675          TGCACCGTCG---ATAAACTTAATACCGGA-TAGAATGAAAAAATACATTTT
BACOVA_05497    TGCACCGTCA---ACGAACTTAATACCAAA-TAGAATGAAGAAAAACATTTT
                **   * *        *  ****** *  *  ** ***** *** *******
```

HTCS_Hya (BT3334) regulon

```
BT3348          TCAAGGGAGAAATGTACCAAATTTGTCCCTGAATTGGATTATTTGGGACGGTCTATTCTATTTGAAAGTACTACTTTTGCC
BACOVA_01969    TTTTCAGTGAAATGTACCAAAATTGTCCTTCATTTGGATTATTTAGGTCGGTCAATTCTATTTAAATGCACTACTTTTGCC
BACFIN_02037    TTCTTTTGCAAATGTACCAAATTTGTCCTTCATTTGGATTATTTAGGTCGGTCAATTCTATTTAAATGCACTACTTTTGCC
Bache_0564      TGATA-TGTAAAAGTAACGATTTTGGCTAATAATTGGATTATTTGTGAAGGTTAATGCTGTTTGCTTGCACTACTTTTGCA
BACCELL_01621   TTATG-TGCAAATGTAACAATTTTGCTCTTTTCCTGTATAATTTAGGAGGGTCTTTTCTGTAAACCTGCGCTACTTTTGCT
BACUNI_01004    TCCGGACGCAAAAGTAACGATTTTAGCCATTAATTGAATTATTTAGGCGGGTCAAATCTGTACACTTGCTCTACTTTTGCA
                *        *** *** * *  **          ** ** ****  *  ***     ** *      *  **********

BT3348          ACC-AGATAATTATTAAC--TTAACAAATAATCCATGTATTATGAAAACAATTC
BACOVA_01969    ATCAGGAAAATTAATCAC--TTAACAAATAATCCCTGTATTATGAAAACAATTC
BACFIN_02037    ATCAAGAAAATTAATCAC--TTAACAAATAACCCGTGTATTATGAAAACAATTC
Bache_0564      GCAGAGAAATAGTAAAACT-TTAATAAATGAATCCTTTATTATGAAAGCAGTCC
BACCELL_01621   GACGAAAGAATACGAAACT-TTAATAAACAAATCAT-TATTATGAAAACAATCC
BACUNI_01004    GCAGGAAAAATCATAAACTCTTAATAAATAAATCCCTTATTATGAAAACAGTCC
                      * *       **  **** ***  *  *   ********** ** * *
```

HTCS_Man-1 (BT2628), HTCS_Man-2 (BT3302), HTCS_Man-3 (BT3786) regulons

```
BT2629         CGGAATATAAGTAAAGCTCCGTGCCAGAGTAGTTAATTTCAGCGACAAGAGGCGATTTAGCCCCTCTATTACTAGAAGC-AC
BACOVA_03629   CAGAATATATGTAAGAATGCGTGCCAGTTTGGTTAATTATAGCGCCACAGGGCGATTTAGTGTTTCGACTTCCGGTAGCGGC
BT3784         CGGAATATAAGTAAAGTTCCGTGCCAGAGTAGTTAACTTCAGCGACAAGAGGCGATTTAGCCCCTCTATTACTAGAAGC-AC
               * ******* ****   * ********  * ***** *  **** **   **********    ** * * *  * ***  *

BT2629         ATTCCTTTTCTCCCTATTTTCGTCGTAGAAATTATTTTCAAATCTATAAATTCATTTGTTCATGAAAACACATTTTT
BACOVA_03629   ATTCCTTTTCTCTCTATTTTCGTCACAGAAACTAATTTTA--TTCATAAATTCATTTGTTCATGAAAACACATTTTT
BT3784         ATTCCTTTTCTCCCTATTTTCGTCGTAGAAATTATTTTCAAATCAATAAATTCATTTGTTCATGAAAACACATTTTT
               ************ ***********  ***** ** *** *  *  ********************************
```

HTCS_Ogl-1 (BT 2826) regulon

```
BACCAC_0055      TAGCATTTTGTGCACCTTATTTGGAGAATATTGCACATCTGTTTTCCACACCTCATCTAACTTTGCAGTATTGTTAAAAGCC
BfaeM_1878       TAGCTATTTGTGCACCTAATTTGGTGAATATTGCACGTCCATTTTTCACATCGTCCCTAATTTTGCATCATCATTAAAAGAT
BT2818           TAGCTATTTGTGCACCTAATTTGGTGAATATTGCACGTCCATTTTTCACATCACCCCTAATTTTGCATCATCATTAAAAGAT
BSIG_1832        TAGCTATTTGTGCACCTAATTTGGTGAATATTGCACGTCCATTTTTCACATCACCCCTAATTTTGCATCATCATTAAAAGAT
                 ****  *********** ****** *********** **  **** **** *    **** ******  **  *******

BACCAC_0055      ACAAATCCATTAATTGTTGACCTTACTTCAAATGAAGCAGA--------TTATTAACTTAACATATTAT-------------
BfaeM_1878       A-------ATGAA--GTTAACCTTATTGCACAT-AAGCAAAGGATATATTTAGAAACTGAAGAGAGAATAAAGAGAATAAAA
BT2818           A-------ATGAA--GTTAACCTTATTGCACAT-AAGCAAAGGATATATTTAGAAACTGAAGAGAGAATAAAGAGAATAAAA
BSIG_1832        A-------ATGAA--GTTAACCTTATTGCACAT-AAGCAAAGGATATATTTAGAAACTGAAGAGAGAATAAAGAGAATAAAA
                 *       ** **  *** ****** * ** ** ***** *        ***  **** ** * *  **

BACCAC_0055      -----------------------------TAATCT---TTCATTATTAACCTAATTAAATTAAACAGTATGAGAAACGCG
BfaeM_1878       GTCACCCCCCAATCCGGTTGACCTAACTATAAACTAAATATATTATTAACCT-ATCAAATTAAACAGTATGAGAAACGCG
BT2818           GTCA-CCCCCAACCCGGTTGACCTAACTATAAACTAAATATATTATTAACCT-ATCAAATTAAACAGTATGAGAAACGCG
BSIG_1832        GTCA-CCCCCAACCCGGTTGACCTAACTATAAACTAAATATATTATTAACCT-ATCAAATTAAACAGTATGAGAAACGCG
                                              *** **   *  *********** ** ************************
```

HTCS_Ogl-2 (BT4137) regulon

1)

```
BT4135           AAAAATTTAATTAACCACAAGATTCATGCTTATTGAAAACACAAGAATGAATTGTTAACACTTTTAATTTCTATGATTAATG
1_1_14_01691     AAAAATTTAATTAACCACAAGATTCATGCTTATCAAAAACACAAGAATGAATTGTTAACACTTTTAATTTCTATGATTAATG
                 *********************************  ***********************************************

BT4135           AAAAACAAAATAGTGGTGGAAGAATACTACAAATATTAATCCTGTTTTGTTTGTATGGTATGTGTTTTACTCCACT
1_1_14_01691     AAAAACAAAATGGTGGTGGAAGAATACTACAATTATTAATCCTGTTTTGTTTGTATGGAATGTGTTCTACTCCACT
                 *********** ******************** ************************* ******* *********

BT4136           TAATTAGTGATTTTTGAAAATAAATTAGTGATTTTTGAAACATACTGCACCATTCATTCTCCTACTTTTGCAGCACACATCA
1_1_14_01692     GAATTAGTGATTTTTGAAAATAAATTAGTGATTTTTGAAACATACCGTGTCCTCCATTCACCTACTTTTGCACAAAACAATT
                  ******************************************** *   * * ***** ************  * ***
```

2)

```
BT4136           AGTTGAATAAATACTCTATAATACATATGAAAAGATTTACACTATTAAATGACCTATCATTTTTCAATTTTTACGATTTGCA
1_1_14_01692     AATCGATTTATTA------AGTATTTATGAAAAAACACAGCCGACATACAAACC------------ACATTTATAGTTCTCA
                 * * ** * * **      * **  ******** *   *  * *   *   ***            *  ****   **  **

BT4136           TTTTCTCTGGATACGAAATA---------------TGCAAATAAG-------AACTTAACATTACCCCAAAAATTCCACTAT
1_1_14_01692     TACTTCTTTGAGATCAATCACCATAACACTATTATTATATATAGGTTTACCAAACTTAACAC--CCCCCTAAGTTCCACGAC
                 *  *   * ** *  **  *               *  * *** *       *********   ****  ** ****** *

BT4136           CTAGCGGCATTG-------GTGCTGGATAGTGGAAATTTTCTCCTAAAAATAGCAATTTCAATTAATACTATGACC--TATG
1_1_14_01692     ----CGGCACCGTCTGACAGTGTCGGCTAGTGGTAACTTATTTAT---------TATTAAAATTAACACCATAAGCATTATG
                     *****  *       ***  ** ****** ** **  *  *          ***  ****** ** ** * *  ****
```

HTCS_Pga (BT4973) regulon

```
BT4672           AGGCATTTTAGGGCTTTTCGTCCTATTTTTATCCTTATATGTCCAATCTGTGCTTTTATGAAAATGCCAGGCAGTCTATCTT
BACOVA_05493     AGGCATTTTGGGGCATTTCGTCCTATTTTTATCCTTATATGTCCAATCTGTGCTTTTATTGAAATGACCAATAAATTACTTT
BACFIN_00640     GGGCATTCTAAAGCATTTCGTCCTATTTTTATCCTTATATGTCCAATCTGTGCTTTTACCTGATTTACCAATAATTTACCTT
                  ****** *   ** *******************************************    * *  *    *   **  **

BT4672           TGCCGCTATCAACAAACCACTAAATTTGATAGCATGAA
BACOVA_05493     TGTTGCTGTCAATAAACCGATAAATTTGATAGCATGAA
BACFIN_00640     TGTCACTATCAATGAACCGATAAATTTGATAGCATGAA
                 **   ** ****  ****  ******************
```

HTCS_Rgu-1 (BT4111/BT4124) regulon

1)

```
BACCELL_03119    GAAAATAAATAATGCACCTTTTCAGTCGAAAAGTACACAACGCTTTAGGTGAGAACCGCTATCTTTGTCACATTACTTAAT
BACSTE_03304     -AAATCAAAAAATGCACCCTCTGCGCCAAAAAGTGCACAGGTGTTTGGAAAGAAACATCTACTTTTGTTTCGTGAAAAATT
BACEGG_00863     -AAATCAAAAAATGCACCCTCTACGCCAAAAAATGCACAGAGGTTTGGAAAGAAACCTCTACTTTTGTTTCATGAAAAACA
BT4108           -AAAACAAAAAATACACCTTCTTAACTAAAAAATCCCACTCTGTTTGGAGGAAATACACTACTTTCGTAGCATGAAAAAAT
BACOVA_04900     -AAAACAAAAAATGCACCTACTTGACTAAAAAATCCCACTGGGTTTGGAGGAAAAACACTACTTTCGTACCGTGAAAAAAG
                  ***  *** *** ****   *      **** * *       *** *     *    ***  ** **  * * *   *

BACCELL_03119    ATAATCTAT-ATACCTAAAAACAAGAACAGAATGATGAACAATCTGCTTTGTAA
BACSTE_03304     ATAAGGTCTAATACCCAAAAAGAACAACTACATGAAAAAGAACTTTATTTGTAA
BACEGG_00863     GTAAAGTCTAATACCAAAAAAGAACATATTCATGAAGAAGAACTTTATTTGTAA
BT4108           AGCATGTATAAT-CCTTAAAACAATAAAC-CATGAGACGAAGCTCTTTTTATAA
BACOVA_04900     ACTATGTTGAAT-CCTTAAAATAACAAAC-CATGAGACGAACTCCTTTTTATAA
                    *  *   ** **  **** ** *     ****     *      *** ***
```

```
2)
BT4114            ----GAAAAACAAAAAAATACTATCGTAAAAACAAATATTACACATCCCACAAGCCCCAAAAACATAATTTTGCACCAGTGA
BACOVA_04911      AACTAAAAAACAAAAAAATACGAGGTGAAAAACAAATATTGCACATCACTATATCTTCATTCTCATACTTTTGTAGCGATGA
BACCELL_03135     ---TAAATAACAAAAAAATACCACCCTAAAAACAGATATTACACATATATATTTTCACATCCCTATACATTTGCAGCAACGA
BACEGG_00870      ---CAGATAACAAAAAAGTACCAACCTAAAAACAAAAATTACACATCTACTTTTTCATATCAATTTACATTTGCAAAAAGGA
                        * ********* *** *    ******* * *** *****            *      **  **** *     **

BT4114            G--AACTCTAAGAGTGGACACACTCGAATATTAATAA------TCTAAT--ATAAAAGAACAAAAGATGCCTAAAGGAATG
BACOVA_04911      ATGAACTCTCATCGGGATTTTTATGAAATATCAAACAACAACCATTAATTAATAAACATGCAAAGAATGTCTAACAAAGTG
BACCELL_03135     ATTAACAGTGTGCCCGAACACACATAACGAA-AAAAAATCATACTTAATTAAT-AACATGCAAAGAATGTCTAACAAAATG
BACEGG_00870      GTTAACAATGTGTAGAGAAACTCATCTGAAT-GAGAAACACTTTTTAAATCATAAATATGCAAAGAATGTCTAA---AATG
                     ***  *                    *   *  *        ***   ** **    ****  *** ****   * **

3)
BfaeM_07678       CCGAATCTAATAACAAAAAAATACAATGTGAAAAACAAATATTGCACACCTGGATATTTTCTTCGTTATACATTTGC-CCCA
BT4116            TTGAATCTAATAACAAAAAAATACAATGTGAAAAACAAATATTGCACACCGACGTCTTTTCTTCCCTATACATTTGCAACCA
BSIG_2528         TTGAATCTAATAACAAAAAAATACAATGTGAAAAACAAATATTGCACACCGACGTCTTTTCTTCCCTATACATTTGCAACCA
1_1_14_01671      TTGAATCTAATAACAAAAAAATACAATGTGAAAAACAAATATTGCACACCGACGTCTTTTCTTCCCTATACATTTGCAACCA
                    ************************************************    * ********  ***********  ***

BfaeM_07678       TAAAATGAACACAATATGTGAATACAACCCTATGAATAAAAAGACTAATGCCATGAATAAA
BT4116            TATAATTAACACAATACGTGAGTACAACCC-ATATTTTAAAGAACTAATGCCATGAATAAA
BSIG_2528         TATAATTAACACAATACGTGAGTACAACCA-ATATTTTAAAGAACTAATGCCATGAATAAA
1_1_14_01671      TATAATTAACACAATACGTGAGTACAACCA-ATATTTTAAAGAACTAATGCCATGAATAAA
                  ** *** ********* **** *******  **   * ***  ******************

4)
BfaeM_07703       TTTCTGTGATAAAATAGTGCACCTAAATGCTCAAAAAGTCCCCTCTCTTTGTATTTGATATAACTTACTTTGC--------
BT4119            TTTTAGTGATAAAATAGTGCACCTAAACGCTCAAAAAGTCCCCTCTCTTTATGTTTTATATAACTTCTTTTGCACATATGA
BSIG_2525         TTTTAGTGATAAAATAGTGCACCTAAACGCTCAAAAAGTCCCCTCGCTTTATGTTTTATATAACTTCTTTTGCACATATGA
1_1_14_01674      TTTTAGTGATAAAATAGTGCACCTAAACGCTCAAAAAGTCCCCTCGCTTTATGTTTTATATAACTTCTTTTGCACATATGA
                  ***  ***************** ***************** **** * *** *********  *****

BfaeM_07703       --------GTAAAACCTTT---------CTGTTAATAGGTGAAAAAGTGA--------GTAACA--------------TTC
BT4119            AACAAAAAATAAGAACTATAAATCAGGAATATTAATGAATAAACATGAGATGATTCCTGTTTCAATTTTCCCCATGTTTTC
BSIG_2525         AACAAAAAATAAGAACTATAAATCAGGAATATTAATGAATAAACATAAGATAATTCCTGTTTCAATTTTCCCCATGTTTTC
1_1_14_01674      AACAAAAAATAAGAACTATAAACCAGGAATATTAATGAATAAACATAAGATAATTCCTGTTTCAATTTTCCCCATGTTTTC
                           *** * ** *          * *****   * ** *   **        **  **              ***

BfaeM_07703       AACT-CTGATTTGTGCCTAACAGATGTTTTGTGAAAAGTATATAGTAAGATGTATTAACTTTAAATTAAGATGGATGAGAA
BT4119            AACGATTGATTTGCATGTAACATAAGTATTG-----ATTAGATAGTAATATGTATTAACTTTAAATTGAAATGAATGAAAA
BSIG_2525         AACGATTGATTTGCATGTAATATAAGTATTG-----ATTAGATAGTAATATGTATTAACTTTAAATTGAAATGAATGAAAA
1_1_14_01674      AACGATTGATTTGCATGTAATATAAGTATTG-----ATTAGATAGTAATATGTATTAACTTTAAATTGAAATGAATGAAAA
                  ***   *******    ***  * * ** ***     * ** ******* ****************** * *** **** **

5)
BfaeM_07723       GAACGACTATTTTTTACTCCTTATCTATCAATATTTGAACTTAATGGTAGTGTACTTCCATTATTTTTGTTCCATTATTTT
BSIG_2521         AAATGACTATTTTTTACTCCTAATCTGTCAATAATTGAACTTAGGGGGAGTAGACTTCCATTACTTTTGTTCCATTACTTT
BT4123            AAATGACTATTTTTTACTCCTAATCTGTCAATAATTGAACTTAGGGGGAGTAGACTTCCATTACTTTTGTTCCATTACTTT
1_1_14_01678      AAATGACTATTTTTTACTCCTAATCTGTCAATAATTGAACTTAGGGGGAGTAGACTTCCATTACTTTTGTTCCATTACTTT
                   **  **************** **** ****** *********  ** ***  ********** ************* ***

BfaeM_07723       TG-------------------------------------------------------------------TCGATAATATAA
BSIG_2521         TGCTTGTGAAATGGGAAAAACATATCAATGAAGATATGGATATTTCTGTAAGTAGTTATCAATAGGAACCCGATAAAATAA
BT4123            TGCTTGTGAAATGGGAAAAACATATCAATGAAGATATGGATATTTCTGTAAGTAGTTATCAATAGGAACCCGATAAAATAA
1_1_14_01678      TGCTTGTGAAATGGGAAAAACATATCAATGAAGATATGGATATTTCTGTAAGTAGTTATCAATAGGAACCCGATAAAATAA
                  **                                                                    ****** ****

BfaeM_07723       ATTTTAAAAAGATGAATACACTTTTGA
BSIG_2521         ATAAAAAAGAGATGAATGGAATTTTGA
BT4123            ATTAAAAAGAGATGAATGGAATTTTGA
1_1_14_01678      ATTAAAAAGAGATGAATGGAATTTTGA
                  **   *** ********  * ******
```

HTCS_Rgu-2 (BT4178/BT4182) regulon

```
1)
BT4172          TTTGTTGAACAAATATCCATTTAAATGAATGATATTCTATTTATCATTCGGGTGGTATTTTCTAATTTTGTGATGTGAAAC
BACOVA_04967    ATAGTTGAACGAATCGCCATATATATGGATAATATTCTATTTCTTAATTGATCGGCTCTCCCTACCTTTGTAAGGTGAG--
BACPLE_00251    ----TTATACAAATTTACAGCAACATGTACAATACTCCAGTCCCTAAGACAGAAGCTTTCCCGTACTTTGTAAAATGAT--
                    **  ** ***   **   * *** *  *** ** * *    *        *   *  *    ***** *  ***

BT4172          CATTGAGAATGTTCCTTATAATGGAACTCCTTATCCTAAATTAGCATATGAAAAGCATAT
BACOVA_04967    -------A---TAGCA---AATATAC-------------------ATATGAAAAAACTCT
BACPLE_00251    -------TATTTTTCC---AATAAAA-------------------CTATGAAGAAACTGT
                           *  *    ***  *                     ****** *   * *

2)
BT4174          TTTCCGAAGGCATTATGGACAAATTTAAATACTTGTGGACAATATTCCATTGAAGAGGAAAAAAGATTCCCCTATCTTTGC
BACOVA_04973    TTTTCTGTCGTCTCGTGAACGAATTTAAAGACATTTGCACGGAAATCCATTGAAAAAGAAAAGAACGTCTCTTACCTTTGC
                *** *    *  *  ** ** ******** ** * ** **   * ********* * ***** *   ** * ** ******

BT4174          CCGTGAAATCAATTATAGTAGCTAATTTTATTTATAAACAGTTTTTATACCATGAAGAAATTACTCCTCTCA
BACOVA_04973    CATAGAAATTAGTATAAACAGCAAAT--TAATAAAACAGAACTATTGTACCATGAAGAAACCACTATTCTTA
                *   ***** * *   *  *** ***  ** * * * * *  * ** *************  ***  *** *

3)
BACOVA_04950    AAGAGCATGGACAATATTTCAAATCCATGTAGTTTTGTACACGAACGCTTTTAGATGTCCATTTTGATACAATATTCTATGT
BT4151          ATGATGATGGAGATATTATCATATTCATGTAGTTTCGTACACCAACCGCCATAAAAGCCGTTTTTGATACAATATTCTATGT
                * **  ***** *   * *** ** ********** ****** ***     ** * * *  *********************

BACOVA_04950    ATCTGTACATCTTTCTATTTACTAAACCCTTAAAAAGCTCTACTTTAGCGGCAGATTTTAAAAAG-AATGAAAAACATATCA
BT4151          ATCTGTACATCTTTCTATTTTATAGATTACTCAAAAGCTCTACTTTAGCAGCAGATTTTTAAACGTAGTGGAAAACATATCA
                ********************  ** *    * ***************** ********* *** * * ** ***********

BACOVA_04950    ACTCTATATATCATGAAACGACTTTTAGTAAGTATTGCTATCGTATTT
BT4151          ATTCTACATATCATGAAACAACTTTTAGTAAGTATTGCCATTGTATTC
                * **** ************ ****************** ** *****

4)
BACOVA_04974    TACGTCTAATGATGATTTCTACATATATCTGTACAATATTCTATTTTATCAATGGGCTGCGTCCTTTACTTTTGT-----G-
BT4175          --GTTAAAAAGGCAATTTATACATAGATTTGTACGATATTCTATTTTATGGGTAGACCATATCGCTTACTTTTGCACGACGT
                    *  ** *   **** ****** ** ***** **************   * * *    **  *********      *

BACOVA_04974    --TTGTAA---------------TTTGATTAATCGATAAAACTATGAAGTGCATAACTCTTTTTTTTAGTTTCT
BT4175          TATTCTAAATATGATAAGAACACTTTGATTAAT-GATAAAACTATGAAATGTACAAGCATCTTTTTTAGTTTGT
                  ** ***               ********** ************** ** * **   * *********** *
```

Crp (BT4338) and AraR (BT0354) regulons

```
                                  Crp                AraR                    AraR
BT0356          TTCTTATCCTTTTATGTTGCACAACATATAAGAGTGTATTTGATACACCAAACAAAAGTGTTACTTTTACACCCAAAA
BACOVA_01715    TACA---CCATTTATGCTGCACAACATATAAGAGTGTATTTGATACACCAAACGAAAGTGTTACTTTTACACCCAAAA
BACUNI_01357    TTTCTTGACACTTATGTTGCATAACATATAAAAGTGTACAGCCTACACTAAAGAAAAGTGTTACTTTTACACCCAAAG
BACEGG_01536    TTTTTTTTCTTTTATGTTACATAACATACAAAAGTGTATATATTACACCAAATAAAAGTGTTACTTTTACACCCGAAA
BACCELL_05604   TTTTTTTACTTTTATGTTACATAACATATACAAGTGTTACTAATACACTCAATAAAAGTGTTACTTTTACACCCAAAA
                *       *  ***** * ** ****** *  *****      *****  **  ******************** **

BT0356          TAAGATAAGCATCTCATTTACATTAAAAATAAAACCATGAAAAAACATTTTCTAATTGCAGGAATTGCCGCGCTGAT
BACOVA_01715    TAAGATAAGCATCTCATTTACATTAAAAATAAAACCATGAAAAAACATTTTCTACTTGCAGGATTTGCCGCGCTGAT
BACUNI_01357    AAAGATAATCATCTCATTTACATTAAATACAAAACCATGAAAAGACATGTTCTACTTGCAGGAATTGCCGCAATGAT
BACEGG_01536    TAAGATAAGAATCTAATTTTCAAATTAAATAACACCATGAAGAATCACATTCTTATTGCCGGAATAGCTACATTAAT
BACCELL_05604   TCGGATAATTATCTCATTTACATTTAAAATAGAACCATGAAAAACAGTTTTCTACTTGCAGGAATTGCCGCACTGAT
                   *****  **** **** **    * * *  ******** *      ****  **** *** * **  *  * **
```

Crp (BT4338) and XylR (BT0791) regulons

```
                                XylR                                              XylR
BACCELL_04758   -CTTCACTCTTATTGTACTTCCTACAATTACCTGATTCTTCCGTTTATCAACAATACATATTGTACTTTCAACGATAAGGCCGT
BACUNI_00695    -CTTTCTCCTTATTGTACTTCCTACAATTAGACCTCTTGTATATTCTTGTAAATTACATATTGTATTCCATACGATAACAACTG
BACSTE_01380    -CTTCATAGTTATTGTATCTGCTACAATTACCCTTGCTGA-GACGGATTTAAATTACATATTGTATTTTATACGATAACACAAT
BACEGG_02642    -GTTTATAGTTATTGTGCCTATTACAATTACAACAATGGAACTTT-ACTTAAATTACATATTGTATTTTGTACGATAACACAAC
BF2354          -TCCCCGGCTTTCTGTACTTTCTACAATTACTATCGTTCACCGAGGGTAGAAA-TACATATTGTTATCGATACAATAAGATGAA
BT0791          TCATTCCGCTTTTTGTACTTTATACAATTAC--TCGTTGCCGGAACGGGTAAAATACATATTGTAATTCAGAAGATAACACTAA
BACOVA_02532    TCCATCTAGTTTTTGTACTTTATACAATTAC--TCACTCGCGGAAATACTAAAATACATATTGTAATTCGTAAGATAACACTAA
                         **  ***   *  ********                    * * **********  *    *  ****
```

```
                             XylR                           Crp
BACCELL_04758   GTTTTGATATTGTAAAATGTACGATATCTATCATATTGGAAAATAGTGCTTTATAACATAATAGAAATTAAGTTTTATGCGCTT
BACUNI_00695    AAAATGATATTGTATAAAGTAAGATATGTATTCAGTTGAAAAATAATGCTTTATAATATTATAAAAAATATTCTTTTCCTTTATT
BACSTE_01380    AAGATGATATTGTAAGATATACGATATGTATATATTTGAAAAATAATGCTTTATAACATTGTAAAAAATATCCTTTTTACTTATT
BACEGG_02642    AAGGTGATATTGTAAAAGATATGATATGTATTTGTCTGTAAAATAATGCTTTGTAACATATCAAAAAATATTCTTTTCGCCTATT
BF2354          TACTCCTTAGTGTAAAATTTACGATATGTATATATATGAAAAACAATGCTTTATAGCATATTA-------TTTATTTGTTTACC
BT0791          GTACTCATATTGTAGATTGTATGATATGTATATCTATGAAAAACAATGTTTTATAGCATGTCA-----TTTGTTATTGTTC---
BACOVA_02532    CTATTCATATTGTAGAATGTATGATATGTATTTATATGAAAAACAATGTTTTATAGCATGTTTCTTATTTTGTTTTGATGGTTT
                  ** ****     ** ***** ***      ** **** * ** *** **  **                *   *

BACCELL_04758   GTATATTCAAAAAACCTGTTTATATTTGCAGCG-CTTAAACA-TGGATATTATGCAAAACATGGAACATAAAACCCCATTAGC
BACUNI_00695    GCAAATTAGAAAAACCTATTTATATTTGCAGCG-CTTAACGA-GATATATTATGCAAAACATAGAACAAAAAACGCCGTTGGC
BACSTE_01380    GCATATTTGAAAAACCTCATTATATTTGCAGCG-CTTAAACG-AAAATATTATGCAAAACGTGGAACAAAATACGCCATTGGC
BACEGG_02642    GCATATTCAAAAAACCTCATTATATTTGCAGCC-GTTAAACA-TAAATATTATGCAAAACATGGAACATAACACGCCATTGGC
BF2354          GCATGAT-----AATATCTCTATATTTGCAGCA-ATCAAAGACTCAATATTATGCATGACATACCCAAACAAATCCCATTGGC
BT0791          ---GAATCATAAAGCGCCCCTATATTTGCATCGTATTAAAGA-AAAGGA-TATGCAAAGTATACAGAAAAAAGCACCCTTGGC
BACOVA_02532    GTAG------------CCTCTATATTTGCATCGTCTTAAAGA-AATGTA-TATGCAAAATTTACAGAAAAATACACCTTTAGC
                                    ********** *   * **        * ******    *     *  *    ** ** **
```

Crp (BT4338) and FucR (BT1272) regulons

```
                                                                 Crp
BT1272          CCTCTTCG-GTTCGCCGAAGGGGATTTTTT--TTATTTCCTGCTATAGAACATATTAAAACAGTTCTCTACTTCTGAATAAATT
BACOVA_03849    CCTCTCCG-GTCCGCCGGAGGGGATTTTTTTATTGTTTTCTGCTATAAAACATATCAAATTATTTATTCCGTTCAGAATAAAAT
BVU_1374        TCTTCCGGAAATCGATATAGAAGCTTCGAT-----TCTTGTGTTATAAAACATACCTTTCATCTTGCTGTTTATGAATAAATT
BACDOR_02265    TCTTCCGGAAATCGATATAGAAGCATCGAT-----TCTTGTGTTATAAAACATACCATTTATCTTGCTGTTTATGAATAAATT
BF0210          ---------AATTGCTTTGT--TCTCTGCC-----TTTGATGTTGTATAACATACCATTTATCTTGCTTATCCAGAATAAACT
                           *                       * *  ** * ** ******      *  *      *   ******* *

                         FucR
BT1272          TCTTTTCTTTGCAACAGCATAGCTCAGCACAGTCATTTATCTTATAACAGTATGAGACGAACTGAT----AAAAAGAAAACATT
BACOVA_03849    GCCTTTCTTTGCATCAGCATAGTTCAGCATAGTGTTATTTATTTTTCGAATATGAAACGAATTGAT----AAAAAGGCAACATT
BVU_1374        GCATTCCTTTGCATCGGGACAGTTCAGCACAG---TTTGCATTGTCCGCGTAAAACCTTAAACAGTTC--ATGAAAGTTACCTT
BACDOR_02265    GCATTCCTTTGCATCGGGACAGTTCAGCACAG---TTTGCATTGTCCGTGTAAAACCTTAAACAGTTC--ATGAAAGTTACCTT
BF0210          ACCTTTCTTTGTATCATGATAGTTCAGCATAGCTATCTACAAT-----TAAATAACCTTTATTGATACGTATGAAAATAACTTT
                 * ** ***** * *   * ** ****** **  * *    *         *  *     *     *    *  **     ** **
```

Crp (BT4338) regulon

```
                                                          Crp
BT4338          TTCGTTCATTTTCTCATTTTTTTCTTTCTACTTTTGTGCTGTATAACACAGTTTTGTAGCA--CCCATGGTAAAAATGAACATGT
BACOVA_05152    TTCGTTCATTTTCTCATTTTTTTCTTTTTACTTTTGTGCTGTATAACACAGTTTTGTAGCA--CTCATGGTAAAAATGAATATGT
BACUNI_04721    TTTGTGCAATATTTAATTTTTTTCTTTTTACATTTGTGCTGTATAACACAGTTTTGCAGCA--GTTATGGTAAAAGTAAACTTAT
BACCELL_03476   TTTGTGCAATATTTAATTTTTTTCTTTTTACATTTGTGCTGTATAACACAGTTTTGCAGCA--GTTATGGTAAAAATGAATTTGT
BACEGG_01340    AATGTATCATTTTTAATTTTTTTCTTTTTACATTTGTGCTGTACAACACAGTTTTGCAGCA--TTTATGGTAAAAATAAACTTAT
BACSTE_03515    AATGTGTCATTTTTAATTTTTTTCTTTTTACATTTGTGCTGTACAACACAGTTTTGCAGCA--TTTATGGTAAAAATAAATTTAT
BF0954          ACTATTCTGTTTTTCATTTTTTTCTTTCTACATTTGTGCTTTATAACACAGTTTTGCAACA--GATATGGTAAAGATGAATTTGT
BACCOPRO_01276  AAAAAGTCGAATTTCATTTTTTTATTCCTATTTTTGTGCTTAATAACACGATTTTGTAACA--CTTATGGTAAAAAAGGAATTAT
BACPLE_02167    AAAAAGAATATTTTCAATTTTTTCTTCGTACCTTTGTGCTTAATAACATGGTTTTGGAACAT-TTTATGGTAAAAAAGGAATTAA
BVU_3580        TTCGTCC----TTTTATTTTTTTCTTCTTATATTTGTGCTCTATAACACGGTTTTATAGCACGTTTATGGTTAGAAAAGAATTAT
BACDOR_01607    TTCGTCC----TTTTATTTTTTTCTTCTTATATTTGTGCTCTATAACACGGTTTTATAGCACGTTTATGGTTAGAAAAGAATTAT
                           * * * ***** **   **   ********  * ****     ****   * **     ***** *        *  *
```
