# Supplementary figures and images for: Polysaccharides utilization in human gut bacterium Bacteroides thetaiotaomicron: comparative genomics reconstruction of metabolic and regulatory networks
Source: BMC Genomics. 2013 Dec 12;14:873. doi: 10.1186/1471-2164-14-873 (PMC3878776; doi:10.1186/1471-2164-14-873)

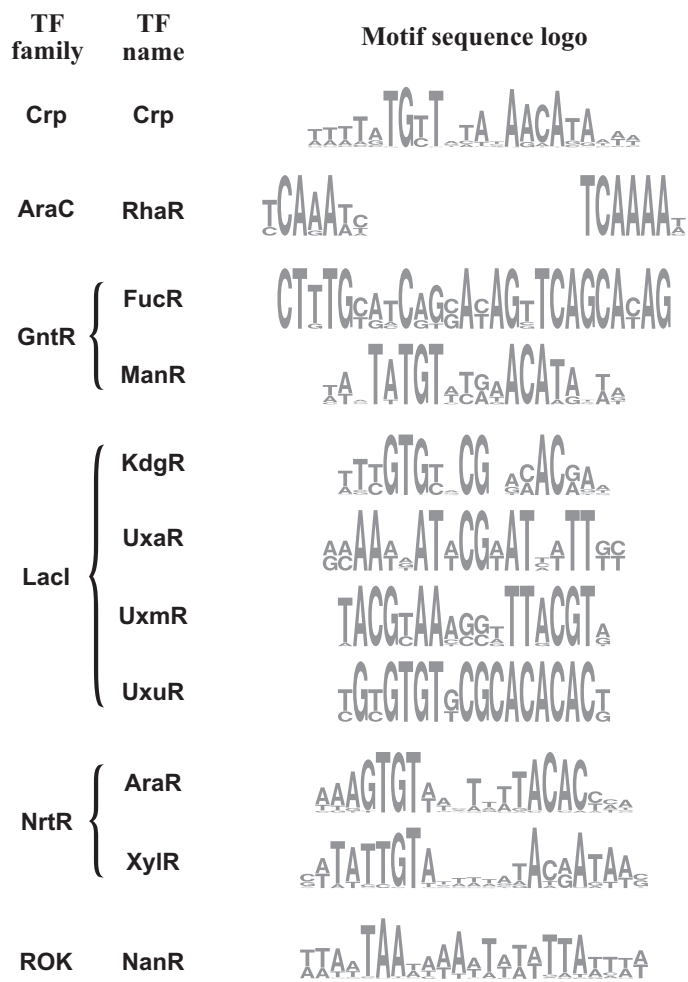

Figure 4

Supplement: Additional file 4: Figure S1 — Maximum-likelihood phylogenetic tree for HTH domains of the HTCS regulators. Proteins with reconstructed regulons are shown in bold. [file 1471-2164-14-873-S4.pdf]
