# Supplementary material for: Polysaccharides utilization in human gut bacterium Bacteroides thetaiotaomicron: comparative genomics reconstruction of metabolic and regulatory networks
Source: BMC Genomics. 2013 Dec 12;14:873. doi: 10.1186/1471-2164-14-873 (PMC3878776; doi:10.1186/1471-2164-14-873)

Reconstructed pathways of polysaccharides utilization in B. thetaiotaomicron.
Genes in the same regulon and regulators for this regulon are shown by the by matching background color.

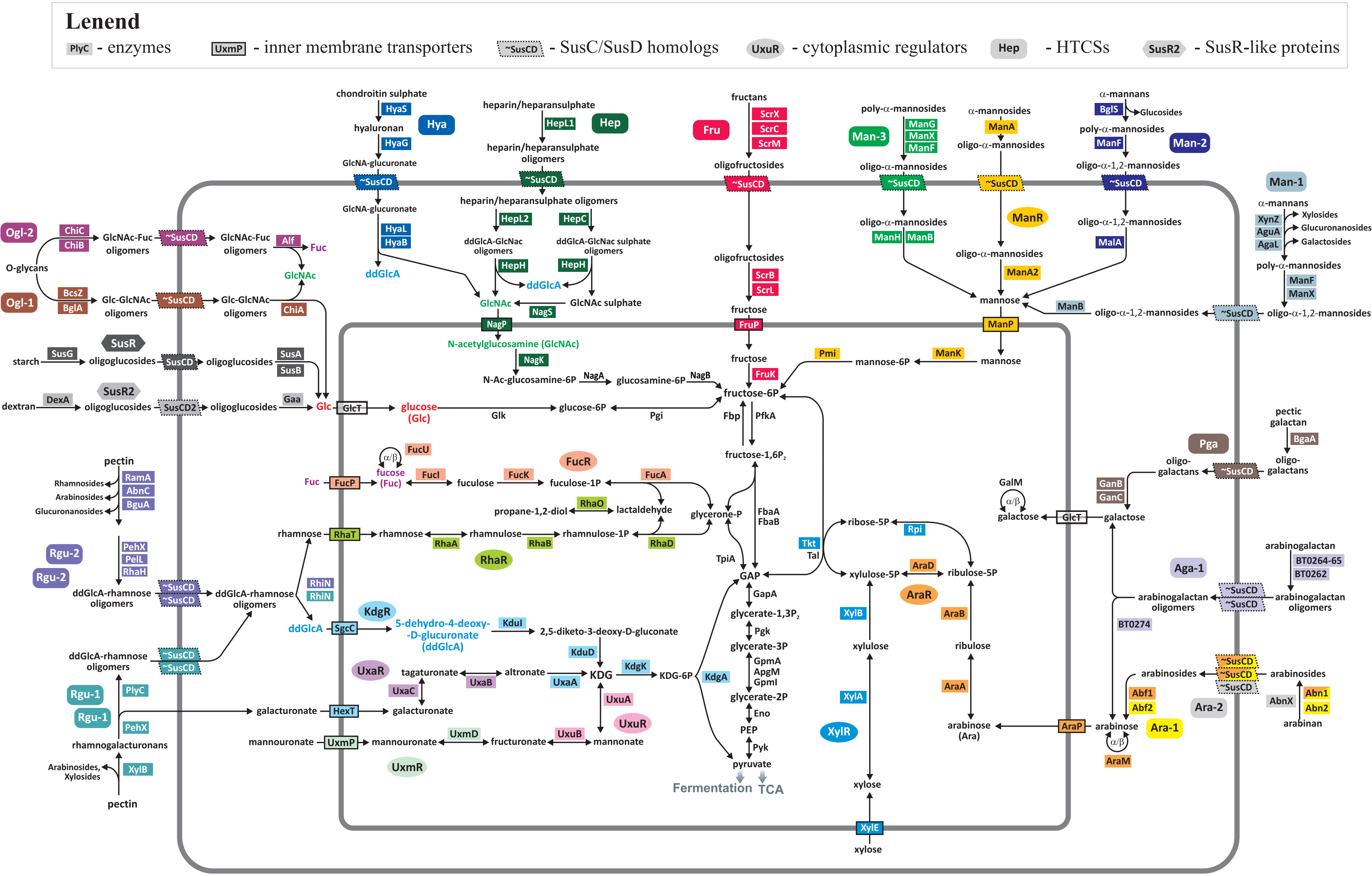

Supplement: Additional file 5 — Reconstructed pathways of polysaccharides utilization in B. thetaiotaomicron. Genes in the same regulon and regulators for this regulon are shown by the matching background colors. [file 1471-2164-14-873-S5.pdf]
